# The immorality of too much money

**DOI:** 10.1093/pnasnexus/pgaf158

**Published:** 2025-06-24

**Authors:** Jackson Trager, Mohammad Atari

**Affiliations:** Department of Psychology, University of Southern California, Seeley G. Mudd Building, 3620 McClintock Ave, Los Angeles, CA 90089, USA; Brain and Creativity Institute, University of Southern California, Seeley G. Mudd Building, 3620 McClintock Ave, Los Angeles, CA 90089, USA; Department of Psychological and Brain Sciences, University of Massachusetts Amherst, 135 Hicks Way, Amherst, MA 01003, USA

**Keywords:** morality, culture, wealth, money, inequality

## Abstract

In some societies, people find excessive wealth immoral, while others are structured so that having too much money is morally neutral or even praised. Here, we show that moral judgments of excessive wealth are distinguishable from moral judgments of economic inequality and examine how people’s moral concerns and national inequality predict the immorality of excessive wealth around the globe. Using demographically stratified samples from 20 nations (N=4,351), we find that across all countries, people do not find excessive wealth very immoral, with notable variability such that more equal and wealthy societies (e.g. Belgium, Switzerland) consider having too much money more wrong. People’s equality and purity concerns reliably predicted their condemnation of excessive wealth, whereas loyalty, authority, and proportionality concerns were negatively associated with condemnation of excessive wealth across societies after controlling for the moralization of inequality, religiosity, political ideology, and demographic variables. We conducted a follow-up study in the United States (N=315), showing that moral purity is more broadly linked to the moralization of *excess* beyond wealth, even after controlling for different ways of wealth acquisition and spending. Collectively, these cross-cultural results demonstrate that some moral intuitions shape our moral judgment of excessive wealth above and beyond economic inequality.

**Significance Statement**

Is having too much money morally wrong? As global inequality widens, examining psychological judgments about wealth is crucial. We analyze generalizable samples from 20 culturally diverse nations and demonstrate that societies with more wealth and equality view excessive wealth as more morally wrong compared with those in unequal less-wealthy societies, suggesting people justify their socioeconomic environment of inequality and may have greater sensitivity to wealth’s harm in wealthier nations. However, across all countries, people do not moralize excessive wealth very much and opinions within nations vary widely. Concerns about moral purity (e.g. cleanliness, naturalness, sanctity) predict the condemnation of excessive wealth, particularly in economically equal societies. We also find a link between moral purity and other types of excess, giving depth to the phrase “filthy rich” beyond merely being an American metaphor. People’s moral concerns and structural economic systems drive moral condemnation of wealth accumulation.

Use your wealth, which is given to you by God at first place, as a mean to please God and secure your life in Hereafter without neglecting your fair share of this world. Be good to the others as God has been good to you and do not spread corruption on earth as God does not like the corrupt people.

Quran, 28:77

For the love of money is a root of all kinds of evil. Some people, eager for money, have wandered from the faith and pierced themselves with many griefs.

Timothy, 6:10

A system that allows billionaires to exist alongside extreme poverty is immoral.

Alexandria Ocasio-Cortez, USCongresswoman

## Introduction

From online bickering about the “filthy rich” to political protests and government policy, the topic of few individuals having an excessive amount of money is a contentious cultural issue. For example, the English proverb “Money doesn’t grow on trees” is a common saying about money, reminding people that it takes hard work to acquire wealth and that money does not come without effort. On the other hand, the Persian proverb “Money is the dirt on the palm of the hand” metaphorically equates money with dirt, emphasizing the unclean nature of wealth and indicating that it ought to be washed away before it corrupts one’s “soul.” Be that as it may, individuals differ substantially in how much wealth they accumulate, and some individuals manage to hoard a disproportionate amount of money: the world’s eight richest individuals have as much wealth as the bottom half of the world.

People’s moral judgment of excessive wealth should not necessarily be the same as their moralization of economic inequality. For example, one might think of economic inequality as morally wrong but still find excessive wealth morally permissible. While inequality is recognized as a moral issue in many societies, it is not clear whether people hold similarly negative views about individuals with excessive wealth. In this research, we investigate the moralization of inequality and the immorality of excessive wealth to explore the extent to which they may represent distinct constructs with potentially unique psychological and cultural underpinnings. The immorality of excessive wealth might vary substantially based on people’s moral intuitions, cultural background, socioeconomic status, and the structural economic systems they live in. As our opening epigraphs suggest, some religions and politicians consider having too much money a root of immorality; hence, religiosity and political ideology can also shape people’s moral views about wealth. In this research, we examine how people’s various moral concerns predict the moral judgment of excessive wealth across 20 nations. We complement this cross-cultural study with a survey in the United States, further examining the relationship between moral concerns and other types of excess (e.g. excessive knowledge, excessive anger) as well as attitudes toward different ways of excessive wealth acquisition and expenditure.

### Psychology of excessive wealth and inequality

In a Pew Research Poll from 2014,^a^ a majority of respondents from all 44 countries surveyed believed that the gap between rich and poor is a big problem facing their country. Why would anyone not condemn inequality in the distribution of wealth? Although most people find economic inequality objectionable, it is less clear if they concomitantly condemn excessive wealth in general. Researchers have found many negative associations with greater inequality, such as reductions in physical and mental health, increased substance abuse, increased violence, lower educational achievement, greater mistrust (1), increased homicide rates (2), decreased solidarity between social strata (3), and decreased happiness (4). However, some argue that inequality and excessive wealth may not be as impending of a social issue as thought, and that the negative downstream consequences are accompanied by less evident but important social benefits (e.g. plunging rates of childhood mortality, world hunger, death from preventable diseases) (see (5)). Similarly, others posit that the negative opinions about inequality may be misguided and based on experimental designs that ignore important considerations such as an individual’s effort in acquiring wealth (6). This consequential disagreement about inequality and extreme nonnormal distributions of wealth around the globe may be rooted in both individuals’ intuitions about right and wrong as well as cultural norms around money, wealth, and deservingness.

Early psychological research with US college students did not find much variance on the topic, showing that in lab settings, the majority of young adults preferred equal distribution of resources. Most participants tended to divide up resources evenly among strangers (7), were committed to this equality even if it meant everyone gets less overall (8), would express anger toward and punish those who distribute unequally (9), and explicitly preferred to live in a more equal society where people at the top (e.g. CEOs) make a considerable amount less than they do now (10). If young American adults prefer equality, then why is this such a contentious topic in US politics? Could there be a deeper cross-cultural explanation for moral judgments of inequality and excessive wealth?

Attempting to address the heterogeneity of opinions about inequality in the lab and in real-life contexts, researchers have made the case that the clear-cut negative opinions found in the lab may be due to poor experimental design that ignores key moral considerations in the real world. Starmans et al. (6) argue that many people actually do prefer unequal distribution of resources, as long as this inequality is an outcome of a “fair” procedure. Drawing upon lab studies, cross-cultural research, and experiments with young children, these authors argue that humans naturally favor *fair* distributions, not equal ones, and that when intuitions about what is truly fair and what is completely egalitarian clash, many people prefer “fair inequality” over equality. These authors argue that if one believes that (i) people in the real world exhibit variation in effort, ability, and moral deservingness, and (ii) a fair system takes these considerations into account, then a preference for this type of fairness will dictate that one should prefer unequal outcomes, including extremely skewed distributions of wealth in the society (6).

Research in social justice has extensively examined this nuanced definition of fairness, differentiating between *distributional fairness*, which refers to equality in outcomes, and *procedural fairness*, which focuses on the equality of procedure (11, 12). Similarly, moral psychologists have conceptualized the fissure in fairness as regulatory concerns about proportionality and equality (13). Studies have demonstrated that the two facets of fairness can prompt varying levels of support for activism that addresses inequality (14). Emotionally, this variation in values is related to the evolved responses to societal free-riders, where people are capable of being both angry at the lazy (which relates to *proportionality*) and also compassionate toward people in need (which relates to *equality*) (15, 16).

Additionally, studies on fairness conducted in the West almost exclusively focus on justice and individual well-being, which are considered Western notions of morality (see (17–19)). Anthropological research outside Western, Educated, Industrialized, Rich, and Democratic (WEIRD (20)) populations suggests that moral values go beyond justice and harm, and usually have notions of hierarchy and purity in them (21, 22). Taking a pluralistic approach to moral values, Moral Foundations Theory (MFT) was developed, arguing that moral cognition is based on our intuitions about at least five foundations: care, fairness, loyalty, authority, and purity (21, 23). More recently, Atari *et al.* (24) split the “fairness” foundation into equality (intuitions about equal treatment and equal outcomes for individuals) and proportionality (intuitions about individuals getting rewarded in proportion to their merit or contribution). According to MFT, a set of intuitions lead humans to “gut-level” judgments of events in the social world (25).

Although Western psychologists have often examined inequality in relation to different flavors of justice (equality vs. proportionality), historically, many philosophical and religious teachings condemn excess regardless of the distribution of wealth in the society because excess corrupts the “soul.” For example, Confucius advocated for exercising self-restraint to avoid extravagances. The founder of Islamic political philosophy, al-Farabi, extensively wrote about the importance of moderation. The Temple of Apollo in ancient Greece had this inscription: “nothing in excess” (26). Moderation and avoidance of excess fall under proscriptive moral regulation, as they protect the *self* (27), have been linked to purity in MFT’s framework (28), and may have evolved to facilitate cooperation through reliable partner selection (29, 30).

We argue that this suggests a less-studied path to understanding excessive wealth: purity. This moral foundation has been argued to be the newest moral intuition in our moral repertoire, possibly culturally evolved with religion (23). As illustrated by our opening epigraphs of excerpts from holy texts, religious teachings may also affect people’s moralization of economic inequality and excessive wealth. Interestingly, while all major religions have explicit cooperative norms about equality, societies marked by high levels of wealth inequality tend to be more religious than those with more egalitarian distributions (31)—which can partially be attributable to higher rates of corruption and nepotism in these societies. This correlation exists across a wide range of countries from different religious traditions and varying levels of economic development. At the individual level, higher levels of religiosity have been found to be associated with the endorsement of the belief in a just world, Protestant work ethic, opposition to equality, right-wing authoritarianism, political conservatism, endorsing the idea that suffering builds character, and other system-justifying beliefs (32, 33). Religions may use the language of purity to tell their followers to stay away from excessive wealth, further facilitating large-scale cooperation (34). Consistent with this view, in many non-WEIRD and more traditional societies, money has been found to be less motivating than psychological incentives (35) and regarded as a *corrupting* element of social life, damaging potential cooperation and degrading one’s “soul” (see (29, 30)). Prior work at the intersection of moral psychology and cultural economics has focused on care, fairness, loyalty, and authority, but has often disregarded purity in explaining economic outcomes (36), especially constructs such as the immorality of excessive wealth—which, we argue, is distinct from the commonly studied judgments about economic inequality.

### Culture, economic inequality, and excessive wealth

Structural and cultural factors play a key role in how our moral intuitions, including those about equality and deservingness, are expressed and acted upon (e.g. (33, 37, 38)). For example, research has shown that the economic system that one comes from may influence one’s opinion toward inequality or excessive wealth (39). This work suggests that indifference to inequality is partly attributable to a belief in the fairness of the capitalist or socialist system in which individuals live. In other words, how a country’s economic system differs with respect to redistribution of wealth may influence citizens’ beliefs about excessive wealth such that individuals from more equally distributive countries (e.g. Belgium) may find excessive wealth more wrong on moral grounds than those from a less distributive sociopolitical system (e.g. Saudi Arabia). A country’s Gini coefficient (i.e. the extent to which the distribution of income or consumption among individuals or households within an economy deviates from a perfectly equal distribution) has been found to be associated with important psychological outcomes such as lower levels of happiness and well-being (40). Even if a nation enjoys substantial economic growth in which the average citizen gets wealthier, the same citizen may not necessarily feel happier if this growth is accompanied by growing inequality as measured by the Gini coefficient (41). When a person’s attention is drawn to their relative standing in the distribution of material well-being, they often exaggerate the impact that personal income has on happiness (42), suggesting that in more unequal societies where one’s relative standing is more salient, one may put more value in their relative income in the face of the excessively rich.

Beliefs about economic inequality and excessive wealth may also be influenced by political ideology (43, 44), even though the psychological mechanisms responsible remain elusive. For instance, American political parties differ on the government’s role: Democrats lean toward more government intervention, while Republicans prefer limited government involvement. In this respect, Republicans may not support government interventions to redistribute wealth to create more equal outcomes and may not regard excessive wealth as a moral issue (see (45)). System Justification Theory (SJT (46)) posits that people are generally motivated to see existing social, economic, and political institutions as fair and thereby enforce the status quo. Thus, beliefs about what is “fair” depend on what is considered to be normative (44). System-justifying beliefs are generally associated with a rightist ideology, belief in a meritocratic system, political conservatism, and right-wing authoritarianism (47).

### The current study

First, we show that the moral judgment of excessive wealth is empirically distinguishable from the moralization of economic inequality. Then, we ask whether the immorality of excessive wealth varies across cultures in predictable ways and whether six moral intuitions conceptualized by a recent theoretical revision of MFT (care, equality, proportionality, loyalty, authority, and purity; (24)) are associated with these judgments across 20 nations. We use MFT to investigate the types of moral intuitions implicated in people’s judgments. Given that WEIRD populations tend to represent both psychological and economic global outliers (48), we recruit participants from a diverse group of nations that varied considerably in their cultural distance and economic institutions (49).

We had three specific predictions based on prior work in cultural and moral psychology and based on MFT (23): (i) Equality should be positively associated with the immorality of excessive wealth across populations; (ii) proportionality should be negatively associated with the immorality of excessive wealth across populations; (iii) purity should be positively associated with the immorality of excessive wealth. Additionally, based on SJT (32) and recent empirical research (38), we predicted that participants from nations with higher wealth equality would find excessive wealth more immoral. Other variables were incorporated for exploratory purposes, including age, gender, religiosity, conservatism, education, GDP, and socioeconomic status.

## Methods

### Participants

We recruited demographically stratified samples mirroring demographics in terms of gender, education, and age (and political ideology in the United States) across 20 nations (Argentina, Belgium, Chile, Colombia, Egypt, France, Ireland, Japan, Kenya, Mexico, Morocco, New Zealand, Nigeria, Peru, Russia, Saudi Arabia, South Africa, Switzerland, United Arab Emirates, and the United States) with 4,351 participants overall. These countries represent substantial variation in terms of cultural distance and wealth inequality. Potential participants were notified of this study by a third-party data-collection platform, Qualtrics Panels, and samples were collected based on the feasibility of stratified data collection. In 14 nations, we recruited 205 participants; in three nations, we recruited 206 participants; in two nations, we recruited 207 participants; and in the United States, we recruited 449 participants. Informed consent was obtained from all participants. University of Southern California’s IRB approved the study (UP-20-00570). Participants were recruited and compensated through a third-party vendor, which ensured that compensation rates were aligned with local wage standards in each country. These data collection was part of a greater survey with additional measures presented elsewhere.

### Measures

All participants first completed a number of surveys, including demographics (gender, age, education, subjective socio-economic status) and other measures of interest described below. All measures were translated into target languages (i.e. Spanish, French, Arabic, Japanese, and Russian) using a third-party professional translation service. Subsequently, independent bilingual researchers checked the translations and verified the fluency of all measures. Discrepancies and modifications were addressed between the translation service, independent researchers, and the second author.

#### Moral foundations questionnaire-2

All participants completed the 36-item Moral Foundations Questionnaire-2 (MFQ-2 (24)), which consists of contextualized items that can gauge moral judgments related to the six moral foundations (i.e. care, equality, proportionality, loyalty, authority, and purity). Items are rated along a 5-point Likert-type scale ranging from 1 (*Does not describe me at all*) to 5 (*Describes me extremely well*) for care, equality, proportionality, loyalty, authority, and purity, respectively. The order of questions was randomized. Internal consistency coefficients (Cronbach’s *α*s) are presented in Table 1.

**Table 1.** Internal consistency coefficients across 20 nations.

Nation	Care	Equality	Proportionality	Loyalty	Authority	Purity
Argentina	0.86	0.84	0.70	0.78	0.77	0.68
Belgium	0.88	0.88	0.68	0.82	0.73	0.68
Chile	0.89	0.81	0.76	0.82	0.82	0.75
Colombia	0.82	0.83	0.72	0.82	0.77	0.74
Egypt	0.85	0.83	0.77	0.82	0.80	0.68
France	0.89	0.86	0.72	0.81	0.73	0.68
Ireland	0.89	0.83	0.80	0.86	0.87	0.77
Japan	0.85	0.82	0.79	0.83	0.77	0.65
Kenya	0.87	0.80	0.76	0.84	0.83	0.73
Mexico	0.86	0.82	0.78	0.79	0.77	0.71
Morocco	0.88	0.83	0.83	0.87	0.80	0.72
New Zealand	0.88	0.87	0.75	0.86	0.86	0.80
Nigeria	0.80	0.83	0.72	0.79	0.72	0.68
Peru	0.84	0.85	0.80	0.81	0.80	0.76
Russia	0.87	0.84	0.74	0.85	0.83	0.74
Saudi Arabia	0.85	0.81	0.77	0.85	0.80	0.65
South Africa	0.84	0.79	0.71	0.82	0.78	0.78
Switzerland	0.88	0.91	0.78	0.85	0.85	0.73
UAE	0.90	0.81	0.89	0.89	0.87	0.78
United States	0.89	0.86	0.72	0.83	0.85	0.77
Average	0.86	0.84	0.76	0.83	0.80	0.72

#### Immorality of excessive wealth

The immorality of excessive wealth was measured by asking the participant to rate on a scale from 1 (*Not wrong at all*) to 5 (*Extremely wrong*), “Is it morally wrong to have too much money?.” Given that distribution of wealth is relative to an individual and cultural context, we chose a generic statement of the ethics of “too much” money instead of an exact number, net worth amount, or percentage.

#### Moralization of economic inequality

Based on prior work in attitude moralization (50), we created a single-item measure to assess how strongly people moralize their attitudes toward economic inequality. To measure participants’ moralization of economic inequality, as a control variable, we asked them “How much are your feelings about inequality based on fundamental questions of right and wrong?” which was rated on a Likert-type scale ranging from 1 (*Not at all*) to 5 (*Very much*).

#### Self-rating of religiosity

Participants then completed a cross-culturally validated single-item measure of religiosity rated along an 11-point scale (0 to 10) asking, ”Generally, how religious are you?” (51).

#### Political ideology

Our working definition of political ideology operationalizes on a basic left-right spectrum to ensure it operates well across national cultures. Participants completed a single-item measure for political ideology (0 to 10) (“In political matters, people talk of *‘the left’* and *‘the right.’* How would you place your views on this scale, generally speaking?”) which can work equally well across cultures (52).

#### Country-level wealth inequality

Country-level inequality was measured using the Gini coefficient, also known as the Gini Index. The coefficient is based on a statistical method that measures how much a country’s income distribution deviates from a perfectly equal distribution. A country with perfect equality in which everyone earns and owns the same amount of wealth has a Gini coefficient of zero while a country with perfect inequality in which one person owns and earns everything would have a Gini coefficient of 100. The benefit of using the Gini coefficient lies in its ability to encapsulate the inequality of the entire income distribution through a single, easily interpretable index that facilitates comparisons between countries, regardless of their population sizes. Our country-level Gini coefficients were gathered from the World Bank Data website.^b^

#### Country-level economic development

Country-level wealth or economic development was measured using Gross Domestic Product (GDP) at purchasing power parity (PPP) per capita. GDP (PPP) per capita is a widely used indicator that reflects the value of all goods and services (calculated by summing consumption, investment, government spending, and net exports) produced within a year divided by the average population and accounting for purchasing power. A higher GDP (PPP) per capita signifies a larger economy and a greater capacity for producing wealth. Our country-level GDP (PPP) per capita data were gathered from the World Bank Data website.^c^

### Analytic procedure

To examine the relationship between moral values and the immorality of excessive wealth, we employed multilevel models to account for the nested structure of our data, which included individuals clustered within countries. Our individual-level variables comprised self-report measures (care, equality, proportionality, loyalty, authority, purity, moralization of inequality) and demographic variables (age, gender [coded 0 for female and 1 for male], subjective socio-economic status, political conservatism, religiosity, and education). Our country-level variables were the Gini coefficient and GDP (PPP) per capita. We standardized all variables and centered all predictor variables at the country level to ensure meaningful comparisons and reduce multicollinearity. For GDP (PPP) per capita, we log-transform the original value to address skewness and nonlinearity and then standardize the logged values to facilitate comparability and interpretation within the model.

We used Gaussian multilevel models and successively added control variables to our base model. Specifically, Model 1 included moral foundations and moralization of inequality. In Model 2, we added demographic variables. Model 3 included the Gini coefficient, and Model 4 further incorporated GDP (PPP) per capita. All models were random-intercept models, allowing for variations in the intercepts across countries, which helps to account for unobserved heterogeneity at the country level. We did not include random slopes as the models did not converge with these parameters, likely due to the complexity of the data and potential overfitting issues. Our model equations were as follows:


$$\begin{array}{rl} {\text{IMOEW}}_{ij} & =\beta_{0}+\beta_{1}\cdot {\text{Individual-level variables}}_{ij-j}\\ & \;+\beta_{2}\cdot {\text{Country-level variables}}_{\;j}\\ & \;+u_{0j}+e_{ij} \end{array}$$

where ${\text{IMOEW}}_{ij}$ is the outcome variable Immorality of Excessive Wealth for individual *i* in country *j*. $\beta_{0}$ is the intercept. $\beta_{1}$ is the coefficient for the combined group-mean centered individual-level predictors: $\left(\;{\text{predictor}}_{ij}-\;¯{\text{predictor}}_{\;j}\right)$. $\beta_{2}$ is the coefficient for the country-level predictors. $u_{0j}$ represents the random intercept for country *j*. $e_{ij}$ is the individual-level error term for individual *i* in country *j*.

Our final model with all individual and country-level factors:


$$\begin{array}{rl} {\text{IMOEW}}_{ij} & =\beta_{0}+\beta_{1}\cdot \text{age}\_\text{std}\_{\text{cent}}_{ij-j}+\beta_{2}\cdot \text{gender}\_\text{std}\_{\text{cent}}_{ij-j}\\ & \;+\beta_{3}\cdot \text{religiosity}\_\text{std}\_{\text{cent}}_{ij-j}+\beta_{4}\cdot \text{conservatism}\_\text{std}\_{\text{cent}}_{ij-j}\\ & \;+\beta_{5}\cdot \text{edu}\_\text{std}\_{\text{cent}}_{ij-j}+\beta_{6}\cdot \text{status}\_\text{std}\_{\text{cent}}_{ij-j}\\ & \;+\beta_{7}\cdot \text{Care}\_\text{std}\_{\text{cent}}_{ij-j}+\beta_{8}\cdot \text{Equality}\_\text{std}\_{\text{cent}}_{ij-j}\\ & \;+\beta_{9}\cdot \text{Proportionality}\_\text{std}\_{\text{cent}}_{ij-j}+\beta_{10}\cdot \text{Loyalty}\_\text{std}\_{\text{cent}}_{ij-j}\\ & \;+\beta_{11}\cdot \text{Authority}\_\text{std}\_{\text{cent}}_{ij-j}+\beta_{12}\cdot \text{Purity}\_\text{std}\_{\text{cent}}_{ij-j}\\ & \;+\beta_{13}\cdot \text{Gini}\_{\text{std}}_{j}+\beta_{14}\cdot \text{MOI}\_\text{std}\_{\text{cent}}_{j}+\beta_{15}\cdot \text{GDP}\_\text{log}\_{\text{std}}_{j}\\ & \;+u_{0j}+e_{ij} \end{array}$$

where ${\text{IMOEW}}_{ij}$ is the outcome variable Immorality of Excessive Wealth for individual *i* in country *j*. $\beta_{0}$ is the intercept. $\beta_{1}$ to $\beta_{12}$ are the coefficients for the group-mean centered individual-level predictors: $\left(\;{\text{predictor}}_{ij}-\;¯{\text{predictor}}_{\;j}\right)$. $\beta_{13}$ to $\beta_{15}$ are the coefficients for the country-level predictors: Gini coefficient, Moralization of Inequality (MOI), and GDP per capita (log-transformed). $u_{0j}$ represents the random intercept for country *j*. $e_{ij}$ is the individual-level error term for individual *i* in country *j*.

The analyses were performed using the “lme4” package, version 4.0.1, in R programming language.^d^ Model fit indices indicated good model fit, supporting the robustness of our findings (see Table 2 for detailed results).

**Table 2.** Multilevel Gaussian models predicting the immorality of excessive wealth.

	Model 1	Model 2	Model 3	Model 4
(Intercept)	0.00	0.01	0.01	0.02
	(0.06)	(0.06)	(0.05)	(0.05)
Care	−0.03	−0.02	−0.02	−0.02
	(0.02)	(0.02)	(0.02)	(0.02)
Equality	$0.33^{a}$	$0.33^{a}$	$0.33^{a}$	$0.33^{a}$
	(0.02)	(0.02)	(0.02)	(0.02)
Proportionality	$-0.09^{a}$	$-0.08^{a}$	$-0.08^{a}$	$-0.08^{a}$
	(0.02)	(0.02)	(0.02)	(0.02)
Loyalty	$-0.08^{b}$	$-0.09^{a}$	$-0.09^{a}$	$-0.09^{a}$
	(0.02)	(0.02)	(0.02)	(0.02)
Authority	$-0.12^{a}$	$-0.11^{a}$	$-0.11^{a}$	$-0.11^{a}$
	(0.02)	(0.03)	(0.03)	(0.03)
Purity	$0.08^{a}$	$0.07^{b}$	$0.07^{b}$	$0.07^{b}$
	(0.02)	(0.02)	(0.02)	(0.02)
Moralization of Inequality	$0.08^{a}$	$0.08^{a}$	$0.08^{a}$	$0.08^{a}$
	(0.01)	(0.02)	(0.02)	(0.02)
Age		$0.07^{a}$	$0.07^{a}$	$0.07^{a}$
		(0.02)	(0.02)	(0.02)
Gender		0.01	0.01	0.01
		(0.02)	(0.02)	(0.02)
Religiosity		0.02	0.02	0.02
		(0.02)	(0.02)	(0.02)
Conservatism		$-0.05^{b}$	$-0.05^{b}$	$-0.05^{b}$
		(0.02)	(0.02)	(0.02)
Education		0.01	0.01	0.01
		(0.02)	(0.02)	(0.02)
Status		$0.04^{c}$	$0.04^{c}$	$0.04^{c}$
		(0.02)	(0.02)	(0.02)
Gini			$-0.13^{c}$	−0.09
			(0.05)	(0.05)
GDP (per capita)				$0.11^{c}$
				(0.05)
AIC	11,481.54	10,834.31	10,831.41	10,829.35
Log Likelihood	−5,730.77	−5,401.16	−5,398.71	−5,396.67
Num. obs.	4,342	4,096	4,096	4,096
Num. groups: country	20	20	20	20

^a^ P<0.001 ; ^b^P<0.01; ^c^P<0.05.

Values outside parentheses represent the coefficient estimate and the values inside parentheses represent the standard error. All predictor variables were centered at the country-level.

## Results

### Descriptive statistics

**Fig. 1.**
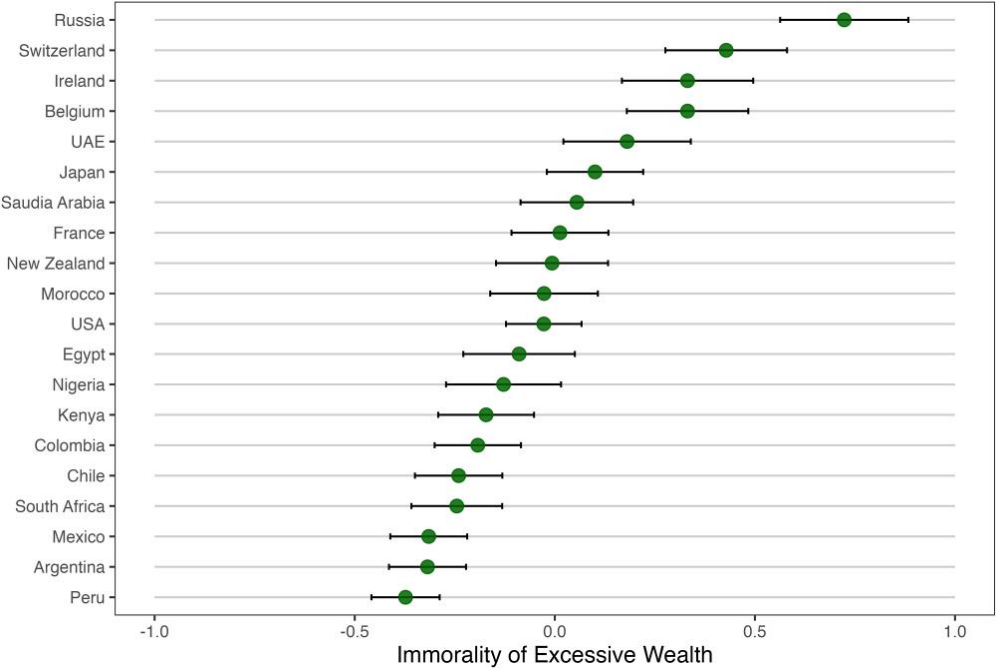
The immorality of excessive wealth in 20 nations. *Note:* Scores are standardized and whiskers represent the 95% CI.

Country-level averages of the moral judgment of excessive wealth are shown in Fig. 1. People in Russia, Switzerland, and Ireland held the strongest moral opposition to having too much money. On the other hand, people in Peru, Argentina, and Mexico were least likely to show moral objections to having excessive wealth. Overall, all national cultures in our study, on average, found excessive wealth to be between “not wrong at all” and “moderately wrong,” indicating that few people might hold the belief that possessing excessive wealth is extremely unacceptable from a moral standpoint.

### Country-level analysis

**Fig. 2.**
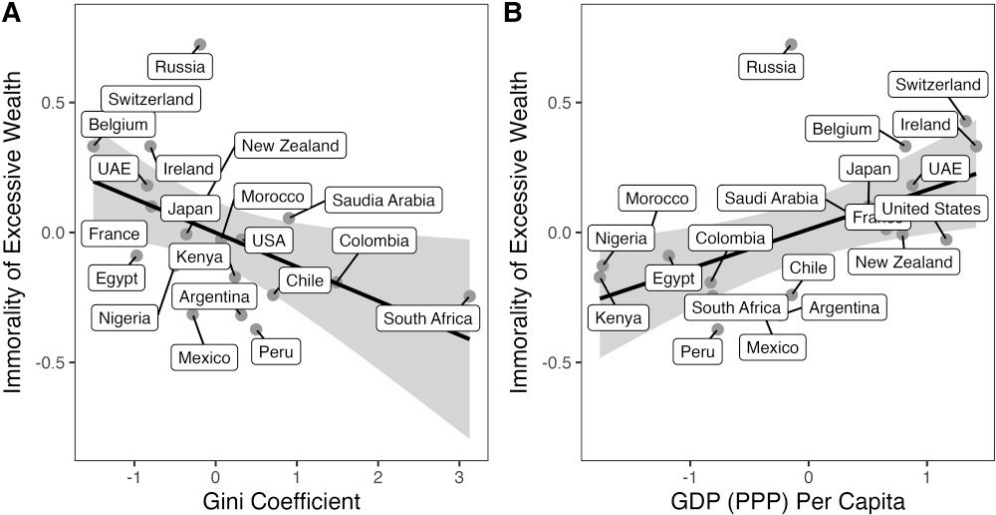
The relationship between the A) Gini coefficient and B) log GDP (PPP) per capita with the immorality of excessive wealth. *Note:* (A) Gini coefficient was standardized where higher numbers represent more inequality and lower numbers represent more equal societies. (B) GDP was log-transformed and then standardized, where higher numbers represent more economic development and lower numbers represent a smaller economy.

The Gini coefficient ($r_{\tau }=-0.43$, P=0.007) and GDP per capita ($r_{\tau }=0.39$, P=0.018) were significantly associated with the immorality of excessive wealth (see Fig. 2) but curiously unrelated to the moralization of inequality (Gini: $r_{\tau }=-0.03,P=0.871$; GDP: $r_{\tau }=0.01,P=0.974$; see Supplementary Materials). Interestingly, Gini had a negative association while GDP per capita had a positive association, suggesting that surrounding income inequality has a different effect than overall level of wealth, and that richer and more equal societies think excessive wealth is wrong, while more developing and unequal societies think it is morally permissible. Countries with lower Gini coefficients (more equal societies like Belgium and Switzerland) were more likely to consider excessive wealth as immoral than countries with higher Gini coefficients (more unequal societies like Peru and Chile). When looking at overall wealth, countries with high GDP per capita (richer countries like Ireland and Switzerland) were more likely to find excessive wealth immoral than countries with lower GDP per capita (Nigeria or South Africa). For robustness checks, we re-ran this analysis while accounting for the nonindependence of nations (53 ), and the results were expectedly weaker (see Supplementary Materials).

### Multilevel modeling

To examine the relationship between moral values and the moral judgment of excessive wealth, we employed multilevel models to account for the nested structure of our data, which included individuals clustered within countries (see Table 2). We standardized all variables and centered all predictor variables at the country level to ensure meaningful comparisons and reduce multicollinearity. All models were random-intercept models, allowing for variations in the intercepts in different countries, which helps to account for unobserved heterogeneity at the country level. We did not include random slopes as the models did not converge with these parameters, likely due to the complexity of the data and potential overfitting issues (see Supplementary Materials for correlation matrices and models without centered predictors as robustness checks).

In Model 1, we entered moral foundation scores as independent variables, and moralization of inequality as a control variable. Consistent with our predictions, equality and purity were positively associated with the moral judgment of excessive wealth, while proportionality showed an inverse relationship with it. All of these three associations held after accounting for demographics (Model 2), country-level Gini coefficients (Model 3), and country-level GDP (PPP) per capita (Model 4). In this final model with all control variables (Model 4), we found that the endorsement of equality (β=0.33,SE=0.02,P<0.001), and purity (β=0.07,SE=0.02,  P=0.002) along with older age (β=0.07,SE=0.02,P<0.001), higher socio-economic status (β=0.04,SE=0.02,P=0.040), the moralization of inequality (β=0.08,SE=0.02,P<0.001), and higher GDP per capita (β=0.12,SE=0.06,P=0.019) to be associated with the moralization of having too much money. On the other hand, Right-wing political ideology (β=−0.05,SE=0.02,P=0.001), proportionality (β=−0.08,SE=0.02,P<0.001), loyalty (β=−0.09,SE=0.02,  P<0.001), authority (β=−0.11,SE=0.03,P<0.001), and the Gini index (β=−0.13,SE=0.05,P=0.015) were found to be associated with moral non-condemnation of excessive wealth.

### Follow-Up Study

The positive association between purity and the immorality of excessive wealth may be considered less intuitive, compared with equality and proportionality. This less intuitive result warranted further investigation to understand purity’s broader relevance to the moral evaluation of excessive wealth.

One possible explanation is that purity concerns about excessive wealth may stem from assumptions about how wealth is *acquired* or *spent*. For example, individuals who are highly concerned with purity might find excessive wealth morally wrong if they believe it is often obtained through exploitative means or spent in selfish or indulgent ways. Additionally, the immorality of excessive wealth may represent a specific instance of a broader relationship between purity and the moralization of *excess* beyond wealth, across various domains. To clarify the role of purity in these moral evaluations, we conducted a follow-up, preregistered study (see Supplementary material) addressing three key questions: (i) How do people feel about different ways of excessive wealth acquisition? (ii) How do people feel about different ways of spending excessive wealth? and (iii) How do people moralize excess in domains other than wealth?

We first ran a pilot study of participants stratified in regards to gender and political orientation to explore if the various ways that people moralize wealth acquisition, wealth spending, and excess beyond wealth cluster into any general categories using exploratory factor analysis with parallel analysis, varimax rotation, and preregistered the follow-up study, see Supplementary material.

For wealth acquisition, we asked about the morality of 10 different paths to acquiring wealth, and our EFA suggested that two factors underlie the variance in these 10 items; these factors were titled “Benevolent Acquisition” and “Exploitative Acquisition.” For wealth spending, we asked about the morality of 10 ways to spend excessive wealth and two factors emerged in the EFA, “Benevolent Spending” and “Exploitative Spending.” For excess beyond wealth, we asked about the morality of 24 different types of excess, which were grouped into four underlying categories, titled “Benevolent Excess,” “Exploitative Excess”, “Constructive Excess,” and “Self Indulgence Excess.” We then used these new factor scores in our Supplementary material.

In order to investigate these questions in a full preregistered study, we recruited a total of 315 US participants, stratified on gender and political orientation. In this study, described in Supplementary material, we asked participants about their moral attitudes on excessive wealth acquisition, excessive wealth spending, excess beyond wealth, and their moral concerns. Our factor analyses suggested that there are two factors underlying wealth acquisition strategies (“benevolent” [e.g. athletics] and “exploitative” [e.g. government corruption]), two factors for spending excessive wealth (“benevolent” [e.g. philanthropy] and “exploitative” [e.g. tax evasion]), and four types of excess (“exploitative” [e.g. patriarchy], “constructive” [e.g. wisdom], “self-indulgence” [e.g. sex], and “benevolent” [e.g. competence]) (for details, see Supplementary Materials).

We found that when controlling for religiosity, conservatism, moral attitudes toward wealth acquisition, moral attitudes toward wealth spending, and other moral values, the endorsement of authority and age still had a significant negative relationship with the immorality of excessive wealth, partially similar to our main study’s findings (see Supplementary Materials). Hence, older people and those high on authority have less severe moral objections to having too much money. Also, the moralization of benevolent spending, exploitative spending, and benevolent acquisition of wealth were all significantly positively associated with the immorality of excessive wealth. The correlation between viewing these specific practices (both acquiring and spending wealth) as immoral and viewing excessive wealth in general as immoral suggests a contextualized moral evaluation that considers both the origins and outcomes of wealth. People seem to morally evaluate not just the existence of excessive wealth, but the paths through which it is obtained and used.

Additionally, in alignment with the main finding from our main cross-country study, we found that purity significantly and positively predicts the moralization of various domains of excess, including benevolent excess (e.g. too much knowledge, happiness, ambition), constructive excess (e.g. too much health, wisdom, friends), and self-indulgence excess (e.g. too much sex, eating, fun). These effects remained robust even after controlling for religiosity, conservatism, other moral concerns, attitudes about wealth spending, attitudes about wealth acquisition, and age (see Supplementary Materials).

This pattern, even though limited to a US sample, suggests that purity’s influence extends beyond traditional religious or political boundaries, shaping moral attitudes about excess across diverse contexts. Purity appears to underlie not only moral judgments of excessive wealth but also broader evaluations of excess, including domains of personal achievement and indulgence.

## Discussion

The world’s richest man in 2023, Elon Musk, said that it is “morally wrong and dumb” to use the word “billionaire” as a pejorative if the individual is using their wealth to create products that are making “millions of people happy.”^e^ His opinion is consistent with the Western tradition that considers happiness maximization the ultimate purpose of morality (54). Is this a cultural universal, however? Many people in our sample appear to agree that having too much money is not immoral, but this view is not universal: left-leaning individuals, people living in egalitarian societies, those who highly value equality, people in higher socio-economic status, and people who value purity appear to think of excessive wealth as more objectionable. These factors are important to consider, as the widespread lack of moral condemnation of excessive wealth may impact efforts to address the societal and ecological harms associated with inequality and overconsumption.

We demonstrated that the immorality of excessive wealth is a distinguishable construct compared with the moralization of economic inequality, with a notably low correlation of r=0.11. Moral concerns and societal factors were differentially associated with the judgment of excessive wealth as immoral after controlling for the moralization of economic inequality. Among these effects, the role of purity is the most interesting since prior work has almost exclusively focused on various interpretations of justice. We also found some cross-cultural evidence that broader structural economic factors may drive moral justification of inequality and money hoarding (see (37)). Specifically, people in wealthier nations tend to regard excessive wealth as more immoral, and the effect of economic inequality (Gini) was substantially reduced after controlling for wealth. This may reflect the greater visibility of harms caused by excessive wealth in wealthier nations, where the effects of hoarded resources and unchecked affluence are more evident. In contrast, individuals in poorer countries may view excessive wealth more favorably, as it is often associated with alleviating basic needs and suffering.

Our findings regarding the relationship between equality, proportionality, and immorality of wealth are intuitively understandable and consistent with prior research (13, 43, 55). The strong relationship between equality concerns and judgments of excessive wealth may partially reflect the wording of several items in MFQ-2, which appear to focus specifically on attitudes toward economic inequality. Moreover, people who highly value the egalitarian distribution of resources in society blame the few who control much wealth and power, and people who believe in a meritocratic system and ideas around effort, the Protestant work ethic, and deservingness tend to see the accumulation of money as morally justified as it corresponds with greater effort, higher creativity, and entrepreneurial activities. The curious finding, however, concerns the relationship between purity concerns and the immorality of too much money (56).

People may feel moral outrage at Billionaires having such amounts of wealth because they find the accumulation of that much wealth corrupting the “soul” of the owner of that wealth, debasing their self-control. As an alternative explanation, it could also be the case that historical levels of corruption (i.e. nepotism) and the absence of strong institutions can shape moral condemnation of excessive wealth. We explored this possibility with further analyses on country-level corruption and the immorality of excessive wealth (see Supplementary Materials), but country-level corruption metrics were not significantly related to the moral condemnation of excessive wealth. A more plausible explanation is that moral concerns explain the relationship between cultural factors (e.g. religion, politics) and the condemnation of excessive wealth.

The soul-degrading nature of money has indeed been highlighted in many cultural traditions and religious teachings (see opening epigraphs), but this finding is not just about abiding by God’s will, as evident in the nonsignificant weak relationship between intrinsic religiosity and the immorality of excessive wealth in our regressions. Individuals who are concerned with purity are sensitive toward corrupting and degrading materials that can infiltrate the sanctity of one’s soul and body (30, 57). These intuitions, based on the present results, are highly correlated with moral opposition to possessing too much money, even after controlling for political ideology and religiosity; hence, billionaires might be considered morally reprehensible, even disgusting, the moral-emotional response strongly associated with purity (58, 59). The purity foundation, as conceptualized by MFT, is related to bodily and spiritual cleanliness, disgust sensitivity, self-control, and avoidance of unnatural things (57–60); thus, people with stronger purity concerns may find having too much money to be impure, disgusting, and unnatural, regardless of how much social disparities money hoarding entails. One potential mechanism explaining this link is self-control and purity-based cooperation. Purity is highly related to self-control (28), and lay theories of excessive wealth entail that extremely rich people get to do everything they want; hence, effectively, there is little inhibiting their impulses. This perceived lack of self-control can lead to judgments of noncooperativeness and immorality (29, 61).

In a follow-up study with a US sample^f^ (62), we found that purity is linked to the moralization of other kinds of excess, including benevolent excess (e.g. excessive happiness, excessive competence). This finding supports the notion that excess, on its own, is considered generally degrading by US adults who highly endorse purity. It could be the case that people who prioritize purity may find moderation (or “mean” of extremes in Aristotelian ethics) morally good, even in things that many strive to have as much as possible (e.g. happiness). Jannoff-Bulman ((26), p. 26) has argued that “the personal strength that serves to ‘protect against excess’ is considered a core virtue,” and our findings suggest that this core virtue mostly falls under the purity foundation. Interestingly, purity’s link to various kinds of excess was robust to different wealth acquisition strategies (benevolent vs. exploitative) and spending (benevolent vs. exploitative).

Notably, an increasing number of economic studies have started relying on MFT to predict economic outcomes. Many of these studies operationalize “moral universalism” as the difference between the two binding, or parochial, moral foundations (i.e. loyalty and authority) and individualizing, or universalist, foundations (i.e. care and fairness) (see (23)), effectively leaving out purity in their equation. For example, in predicting voting patterns and economic outcomes based on moral foundations, Enke intentionally “ignored [purity] because ‘divine’ values are not directly related to the distinction between universalist and communal ones” ((36), p. 3690). The present work highlights the important role of moral purity in predicting economic outcomes even after controlling for all other moral foundations, socio-economic status, religion, political ideology, and country-level economic inequality. As such, we encourage future research in cultural economics to incorporate purity concerns in modeling economic outcomes.

### Limitations and future directions

This research has a number of limitations to be addressed in future work. This research is observational; experimental approaches should be conducted, possibly by framing excessive wealth with certain moral foundations prior to measuring the immorality of excessive wealth (see (63)), in order to establish a causal relationship between these constructs. Additionally, structural factors such as the Gini coefficient may influence the immorality of excessive wealth in longer time periods, so longitudinal or historical–psychological studies are encouraged to study these interactions between economic systems and human psychology temporally (see (64)). Thirdly, the relatively small correlation between the two constructs of “the immorality of excessive wealth” and “moralization of inequality” may suggest they are distinct; however, this observed difference could also be attributed to variations in the phrasing of the survey items (i.e. one addresses the moral basis of feelings, the other directly assesses moral wrongness). Furthermore, the use of single-item measures for key variables, while common in large-scale cross-cultural surveys where space for lengthy assessments of each concept is constrained, may limit the robustness of the findings and should be supplemented with multi-item scales in future research (65–70). Future studies might explore how participants interpret our dependent variable asking about “too much money” or test robustness to alternative wordings, such as “Is it morally wrong to have more money than you need to live a comfortable lifestyle?” or “Is it morally wrong to have much more money than other people in your country?” Much of people’s contemporary moral views on social issues, including the nonnormal distribution of wealth, can have historical roots in their society (71). Finally, some authors have argued that the concept of purity has revolutionized moral psychology, indicating that it may be multifaceted (72). It is a worthwhile future direction to examine the relationships between different facets or meanings of purity and the typology of excess that we developed in our Supplementary material.

## Conclusion

Why do some people deem having too much money to be morally wrong? While systems of faith and systems of government differ in their ethical stance on the cultural issue, given the results of this study, an individual’s moral intuitions and their cultural milieu shape people’s moral judgments of excessive wealth, even if acquired by honest means. Moral condemnation of excessive wealth is not just about harm or different flavors of justice; rather, it may have a more complex moral underpinning. To many, possession of excessive wealth may be disgusting and unnatural due to the degrading nature of excess, suggesting there is more of a psychological truth to the term *filthy rich* than merely being an American metaphor. Our findings have implications for understanding the relationship between moral intuitions and the economic and cultural systems that shape attitudes about excessive wealth beyond concerns for economic inequality.

## Supplementary Material

pgaf158_Supplementary_Data

## Data Availability

The data underlying this article and R scripts are available at our Open Science Framework (OSF) https://osf.io/jkceu/.

## Notes

a
https://www.pewresearch.org/global/2014/10/09/emerging-and-developing-economies-much-more-optimistic-than-rich-countries-about-the-future/

b
https://data.worldbank.org/indicator/SI.POV.GINI

c
https://data.worldbank.org/indicator/NY.GDP.MKTP.CD

d
https://cran.r-project.org/web/packages/lme4/index.html

e Elon Musk, 2022 May 27, 4:16 PM, https://twitter.com/elonmusk/status/1529961091656212514.

f We do not refer to this study as a “replication,” because theoretically, to truly replicate our cross-cultural finding, we would need another cross-cultural sample.

## Preprint

This manuscript was published in a preprint: https://osf.io/preprints/psyarxiv/a7gbk.
