# Supplementary material for: The immorality of too much money
Source: PNAS Nexus. 2025 Jun 24;4(6):pgaf158. doi: 10.1093/pnasnexus/pgaf158 (PMC12199241; doi:10.1093/pnasnexus/pgaf158)
Supplement: pgaf158_Supplementary_Data [file pgaf158_supplementary_data.pdf]

The Immorality of Too Much Money

Jackson Trager, Mohammad Atari

1 Supplementary Information

In this document, we present supplementary and additional analyses related to the main text. All of the following analyses were completed in R version 4.0.1 with the following packages: "haven" [27], "Hmisc"[9], "readxl"[24], "tidyverse"[25], "psych"[18], and "dplyr"[26] for data management, "lme4"[3], "lavaan"[19], "brms" [4] and "lmerTest"[10] to run country-level, multilevel, and mediation analyses, "modelsummary"[1], "ggplot2" [23], "ggside"[11], "sjPlot" [14], "jtools" [12], "xtable" [13], "apaTables"[20], "corrplot"[22], "pwr" [5] for power analysis, "plotrix"[8] and "ggridges" [28] for plots, graphs, tables, and data visualizations, and "glmmTMB"[15] for fitting generalized linear mixed models. All R scripts are available at our Open Science Framework (OSF) https://osf.io/jkceu/.

In the Supplementary Materials, we first provide additional analyses and visualizations on the data from the studies reported in the main text (on the immorality of excessive wealth and country-level inequality and GDP) in the form of descriptive statistics, correlation matrices of variables, and additional Gaussian analysis as robustness checks. We then provide an exploratory analysis of the role of country-level corruption, an alternative factor that may influence how people moralize having too much money across cultures.

Note: The quote from the Quran used in the beginning our main paper is a direct qoute from "An Easy to Understand Translation of Qur'an Paperback" – January 13, 2005 by Bijan Moeinian[17].

1.1 Additional Analysis on Main Study Data

Here, we provide additional analysis and data visualizations for the main study data on the relationship between country-level inequality, moral foundations, and the immorality of excessive wealth.

1.1.1 Country-level Descriptive Statistics

Table S1 Descriptive Statistics by Country: Six Moral Foundations + Immorality of Excessive Wealth + Moralization of Inequality

Country	IMOEW	MOI	Care	Eq	Prop	Loy	Auth	Purity
Argentina	1.32 (0.75)	3.14 (1.09)	3.84 (0.77)	2.81 (1.01)	3.91 (0.66)	3.58 (0.82)	3.67 (0.73)	2.60 (0.82)
Belgium	2.01 (1.18)	3.19 (1.06)	3.91 (0.73)	3.20 (0.94)	3.91 (0.56)	3.62 (0.77)	3.70 (0.64)	3.01 (0.74)
Chile	1.40 (0.85)	3.34 (1.07)	3.77 (0.82)	2.77 (0.88)	3.70 (0.69)	3.45 (0.88)	3.67 (0.81)	2.54 (0.85)
Colombia	1.46 (0.84)	3.05 (1.18)	3.83 (0.71)	2.91 (0.90)	3.69 (0.68)	3.67 (0.82)	3.84 (0.68)	2.98 (0.86)
Egypt	1.57 (1.09)	3.37 (1.04)	4.38 (0.60)	3.56 (0.94)	4.37 (0.58)	4.42 (0.62)	4.18 (0.68)	4.19 (0.63)
France	1.67 (0.95)	3.19 (1.13)	4.08 (0.68)	3.23 (0.92)	4.12 (0.54)	3.86 (0.74)	3.88 (0.62)	3.09 (0.74)
Ireland	2.01 (1.28)	3.58 (1.07)	4.01 (0.79)	2.94 (0.93)	3.73 (0.77)	3.29 (0.98)	3.49 (0.91)	2.51 (0.93)
Japan	1.77 (0.94)	2.75 (0.90)	3.03 (0.77)	2.27 (0.78)	3.14 (0.73)	2.66 (0.82)	2.67 (0.66)	2.63 (0.69)
Kenya	1.48 (0.93)	3.78 (1.18)	4.20 (0.77)	2.88 (0.97)	3.78 (0.79)	3.95 (0.90)	4.07 (0.80)	3.58 (0.83)
Mexico	1.33 (0.75)	3.17 (1.04)	3.77 (0.79)	2.87 (0.91)	3.80 (0.70)	3.78 (0.75)	3.94 (0.67)	2.81 (0.81)
Morocco	1.63 (1.04)	3.08 (1.10)	4.21 (0.78)	3.36 (0.97)	4.18 (0.71)	4.16 (0.82)	3.95 (0.76)	3.93 (0.73)
New Zealand	1.65 (1.09)	3.32 (1.13)	3.84 (0.78)	2.61 (1.02)	3.61 (0.71)	3.22 (1.00)	3.48 (0.87)	2.58 (0.98)
Nigeria	1.52 (1.12)	3.98 (1.00)	4.32 (0.64)	2.90 (1.03)	4.14 (0.67)	4.11 (0.74)	4.21 (0.61)	3.80 (0.77)
Peru	1.26 (0.66)	3.14 (0.95)	3.62 (0.73)	2.63 (0.92)	3.75 (0.69)	3.73 (0.76)	3.81 (0.69)	3.00 (0.82)
Russia	2.43 (1.25)	3.36 (1.08)	3.96 (0.75)	3.24 (0.87)	4.27 (0.48)	3.87 (0.81)	3.68 (0.76)	3.25 (0.80)
Saudi Arabia	1.72 (1.10)	3.22 (1.03)	4.24 (0.75)	3.32 (0.93)	4.18 (0.69)	4.20 (0.78)	4.07 (0.73)	3.98 (0.72)
South Africa	1.40 (0.88)	3.64 (1.13)	4.21 (0.69)	3.01 (0.92)	4.03 (0.64)	3.85 (0.86)	4.00 (0.73)	3.40 (0.94)
Switzerland	2.12 (1.18)	3.22 (1.16)	3.95 (0.68)	3.27 (0.98)	3.84 (0.64)	3.58 (0.85)	3.52 (0.81)	2.95 (0.79)
UAE	1.85 (1.24)	3.29 (1.07)	4.01 (0.92)	3.28 (0.93)	3.96 (0.89)	4.02 (0.91)	3.91 (0.89)	3.74 (0.85)
United States	1.63 (1.09)	3.44 (1.18)	4.04 (0.80)	2.56 (1.03)	3.72 (0.73)	3.50 (0.97)	3.72 (0.89)	2.70 (0.98)

Table S2 Descriptive Statistics by Country: Demographic Variables

Country	Age	Gender	Religiosity	Conservatism	Education	Status
Argentina	42.51 (14.97)	0.52 (0.52)	4.20 (3.13)	5.66 (2.49)	3.36 (1.37)	6.00 (1.67)
Belgium	45.05 (17.01)	0.51 (0.51)	4.53 (3.33)	5.58 (2.20)	3.17 (1.35)	6.02 (1.52)
Chile	42.44 (16.16)	0.52 (0.52)	4.19 (3.03)	5.62 (2.45)	4.17 (1.54)	5.86 (1.52)
Colombia	40.98 (14.98)	0.53 (0.53)	5.46 (3.09)	5.57 (2.77)	3.92 (1.56)	5.34 (1.86)
Egypt	44.84 (16.78)	0.51 (0.51)	7.48 (2.00)	5.24 (2.87)	3.93 (0.65)	6.86 (1.88)
France	43.66 (16.86)	0.52 (0.53)	4.10 (3.22)	5.74 (2.40)	2.93 (1.32)	5.78 (1.75)
Ireland	44.81 (16.72)	0.50 (0.50)	3.92 (3.23)	5.53 (2.03)	3.26 (1.22)	5.71 (1.58)
Japan	47.18 (15.35)	0.52 (0.53)	3.27 (2.74)	5.50 (1.72)	3.35 (1.25)	5.51 (1.88)
Kenya	37.62 (12.42)	0.53 (0.52)	7.39 (2.33)	5.58 (2.96)	3.62 (0.93)	5.68 (2.23)
Mexico	41.91 (15.39)	0.56 (0.54)	5.60 (2.97)	5.78 (3.10)	3.70 (1.05)	6.38 (1.56)
Morocco	41.82 (14.71)	0.54 (0.54)	7.21 (2.00)	5.30 (2.89)	3.51 (1.24)	6.39 (2.13)
New Zealand	47.37 (18.21)	0.54 (0.54)	3.57 (3.44)	5.51 (2.13)	3.29 (1.15)	5.96 (1.66)
Nigeria	39.13 (13.63)	0.59 (0.50)	7.34 (2.40)	5.82 (2.97)	3.97 (0.81)	6.09 (2.17)
Peru	37.05 (13.78)	0.63 (0.49)	5.87 (2.55)	6.59 (2.78)	3.95 (1.36)	6.16 (1.73)
Russia	41.66 (14.87)	0.58 (0.56)	4.35 (3.08)	5.84 (2.47)	3.64 (1.22)	5.33 (1.74)
Saudi Arabia	42.39 (15.48)	0.53 (0.53)	7.47 (2.07)	5.47 (2.92)	3.62 (0.93)	6.46 (2.13)
South Africa	41.31 (15.40)	0.56 (0.55)	6.11 (3.05)	5.98 (2.47)	2.93 (0.99)	5.13 (1.98)
Switzerland	46.74 (16.85)	0.50 (0.50)	4.57 (3.27)	5.48 (2.20)	3.42 (1.43)	6.06 (1.84)
UAE	43.06 (14.67)	0.52 (0.52)	7.16 (2.31)	5.20 (2.85)	4.10 (0.92)	7.01 (2.18)
United States	46.91 (17.87)	0.49 (0.50)	4.94 (3.29)	3.36 (1.52)	3.24 (1.06)	5.53 (1.96)

Table S3 Descriptive Statistics by Country: Gini, GCI, and GDP

Country	GDP (PPP) per cap	Gini	Protestantism %
Belgium	49927.00	27.40	1.35
Chile	15356.00	44.40	13.00
Colombia	6624.00	50.40	12.50
Egypt	4295.00	31.50	2.00
France	40886.00	31.60	2.00
Ireland	103983.00	32.80	4.20
Japan	34017.00	32.90	0.40
Kenya	2099.00	40.80	60.80
Mexico	11497.00	36.80	7.30
Morocco	3442.00	39.50	0.02
New Zealand	48419.00	36.20	26.70
Nigeria	2163.00	35.10	37.70
Peru	7126.00	42.80	12.50
Russia	15271.00	37.50	2.00
Saudi Arabia	30448.00	45.90	3.33
South Africa	6767.00	63.00	72.90
Switzerland	93260.00	32.70	27.00
UAE	53708.00	32.50	5.00
United States	76330.00	41.40	43.00

Fig. S1 *The Immorality of Excessive Wealth in 20 Nations Ridge Plot.*

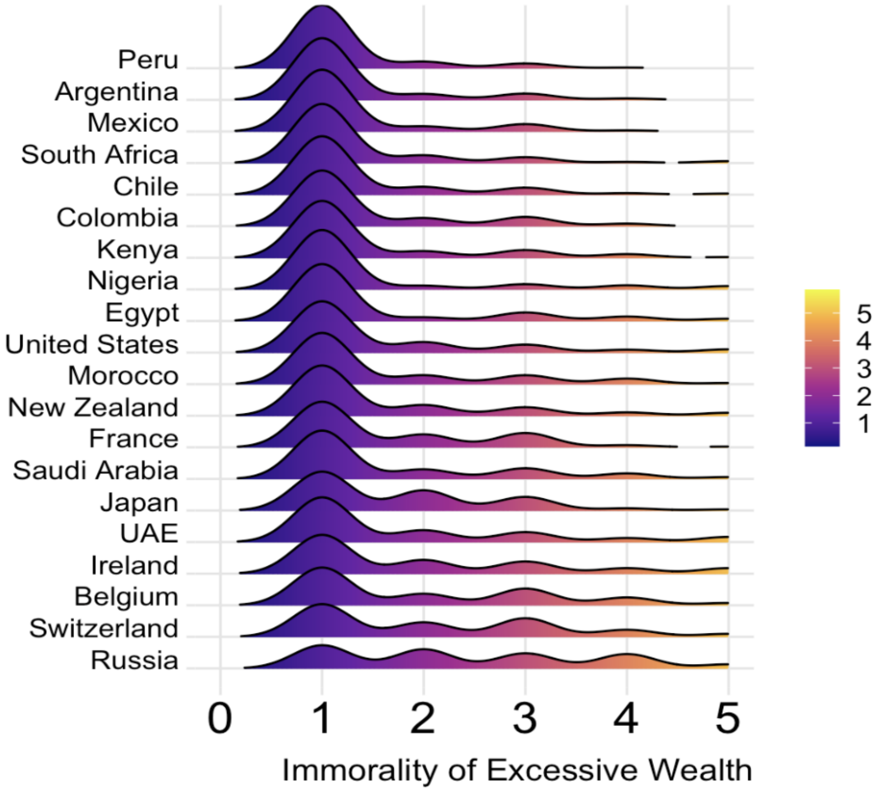

1.1.2 Correlation Matrices

Below are Pearson correlation matrices (Table S4 and Figure S2) of all relevant variables, including the six moral foundations, the immorality of excessive wealth (MOEW), moralization of inequality (MOI), Gini coefficient, GDP per capita, GCI, Protestantism, and demographic measures. It is important to note that without a multi-level model that controls for country-level factors, all variables except for the endorsement of care and religiosity have significant relationships with our dependent variable, i.e., MOEW (see the first column on the left in both matrices).

Fig. S2 *Correlation Matrix Plot of All Relevant Variables.*

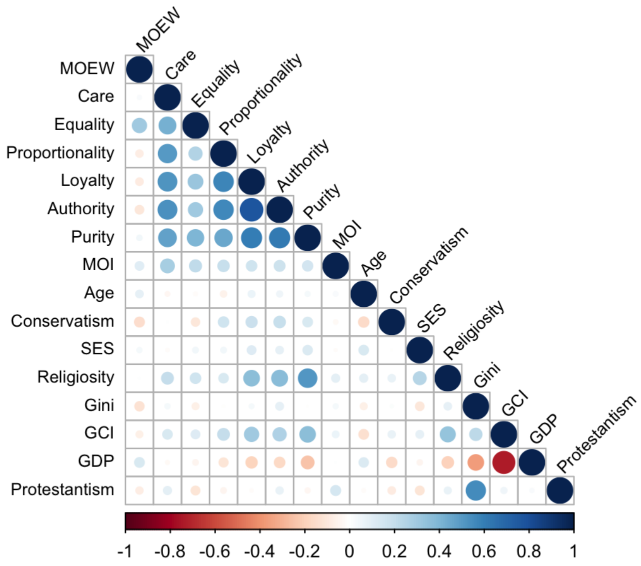

Note:In the shaded row, each cell contains a circle shaded blue or red depending on the sign of the correlation, and with the intensity of color scaled 0 to 100% in proportion to the magnitude of the correlation. MOEW = Immorality of excessive wealth, MOI = Moralization of inequality, SES = Socio-economic status, Gini = Gini Coefficient.

Table S4 Correlation Matrix of All Relevant Variables

	MOEW	Care	Eq	Pr	Loy	Auth	Pur	MOI	Age	Cons	SES	Rel	Gini	GCI	GDP	Prot
MOEW	1.00															
Care	0.02	1.00														
Eq	0.30***	0.43***	1.00													
Pr	-0.08***	0.50***	0.26***	1.00												
Loy	-0.08***	0.53***	0.33***	0.58***	1.00											
Auth	-0.11***	0.53***	0.30***	0.57***	0.77***	1.00										
Pur	0.04**	0.47***	0.40***	0.45***	0.61***	0.61***	1.00									
MOI	0.11***	0.28***	0.21***	0.19***	0.16***	0.17***	0.16***	1.00								
Age	0.08***	-0.03	-0.02	-0.06***	0.07***	0.05***	0.03*	0.03	1.00							
Cons	-0.14***	0.00	-0.10***	0.16***	0.18***	0.20***	0.14***	-0.03	-0.16***	1.00						
SES	0.04*	-0.02	0.05***	0.05***	0.11***	0.08***	0.13***	-0.01	0.13***	-0.00	1.00					
Rel	0.00	0.21***	0.16***	0.13***	0.36***	0.38***	0.51***	0.09***	0.09***	0.08***	0.25***	1.00				
Gini	-0.13***	0.04*	-0.07***	-0.01	0.03*	0.09***	-0.00	0.03**	-0.07***	0.01	-0.11***	0.08***	1.00			
GCI	−0.07***	0.13***	0.11***	0.20***	0.30***	0.26***	0.36***	0.06***	−0.14***	0.08***	0.09***	0.34***	0.22*	1.00		
GDP	0.14***	-0.02	−0.04***	−0.12***	−0.19***	−0.16***	−0.24***	0.00	0.13***	−0.16***	−0.04**	−0.20***	−0.36***	−0.73***	1.00	
Prot	−0.08***	0.09***	−0.11***	−0.04***	-0.01	0.08***	−0.04*	0.14***	-0.02	−0.08***	−0.11***	0.07***	0.56***	0.05***	0.02	1.00

*** $p < 0.001$; ** $p < 0.01$; * $p < 0.05$

Note: MOEW = Immorality of excessive wealth, Eq = Equality, Pr = Proportionality, Loy = Loyalty, Auth = Authority, Pur = Purity, MOI = Moralization of inequality, Cons = Conservatism, SES = Socio-economic status, Gini = Gini Coefficient, GCI = Global Corruption Index, GDP = Gross Domestic Product Per Capita, Protest = Protestant Population Percentage.

1.1.3 Additional Models with Standardized But Not Group Mean Centered Predictors

Table S5 Multi-level Gaussian Models Predicting the Immorality of Excessive Wealth with Standardized But Not Group Mean Centered Predictors

	Model 1	Model 2	Model 3	Model 4
(Intercept)	-0.01	0.01	0.00	0.01
	(0.05)	(0.05)	(0.05)	(0.04)
Care	-0.03	-0.02	-0.02	-0.02
	(0.02)	(0.02)	(0.02)	(0.02)
Equality	0.34^{***}	0.33^{***}	0.33^{***}	0.33^{***}
	(0.02)	(0.02)	(0.02)	(0.02)
Proportionality	-0.09^{***}	-0.08^{***}	-0.08^{***}	-0.08^{***}
	(0.02)	(0.02)	(0.02)	(0.02)
Loyalty	-0.08^{**}	-0.09^{***}	-0.09^{***}	-0.09^{***}
	(0.02)	(0.02)	(0.02)	(0.02)
Authority	-0.12^{***}	-0.12^{***}	-0.12^{***}	-0.12^{***}
	(0.02)	(0.03)	(0.03)	(0.03)
Purity	0.08^{***}	0.07^{**}	0.07^{**}	0.08^{***}
	(0.02)	(0.02)	(0.02)	(0.02)
Moralization of Inequality	0.08^{***}	0.08^{***}	0.08^{***}	0.08^{***}
	(0.01)	(0.02)	(0.02)	(0.02)
Age		0.07^{***}	0.07^{***}	0.07^{***}
		(0.02)	(0.02)	(0.02)
Gender		0.01	0.01	0.01
		(0.02)	(0.02)	(0.02)
Religiosity		0.02	0.02	0.02
		(0.02)	(0.02)	(0.02)
Conservatism		-0.05^{**}	-0.05^{**}	-0.05^{**}
		(0.02)	(0.02)	(0.02)
Education		0.01	0.01	0.01
		(0.02)	(0.02)	(0.02)
Status		0.04^{*}	0.04^{*}	0.04^{*}
		(0.02)	(0.02)	(0.02)
Gini			-0.09	-0.05
			(0.05)	(0.05)
GDP				0.10^{*}
				(0.05)
AIC	11474.46	10826.98	10825.65	10823.51
Log Likelihood	-5727.23	-5397.49	-5395.82	-5393.75
Num. obs.	4342	4096	4096	4096
Num. groups: country	20	20	20	20

$^{***}p < 0.001$; $^{**}p < 0.01$; $^{*}p < 0.05$

Note: Values outside parentheses represent the coefficient estimate and the values inside parentheses represent the standard error. All predictor variables were centered at the country-level.

1.1.4 Additional Models with Country-Level Protestantism

In an exploratory analysis, we asked whether country-level Protestantism plays a unique role in explaining why people feel that excessive wealth is wrong or not. This was inspired by how the protestant church historically has been less negative toward accumulating wealth, even to the point of John Calvin writing about how usury is not *necessarily* wrong, and is permissible if it is not used for immoral ends [21]. This is in stark contrast to the ban on usury in both the Catholic church and Islam (both Sunni and Shia). Usury in Arabic is translated to the word "*Riba*," which also literally means "excess". Therefore, we explored whether the link between excessive wealth and purity we found in our main study should be depressed in countries that have high levels of Protestantism. In order to examine this relationship, we investigated the interaction between country-level Protestantism and purity. We gathered country-level Protestantism[1] and ran additional multi-level models.

To establish a baseline understanding of the relationship between purity and beliefs about excessive wealth while controlling for Protestantism and other factors, we added Country-level Protestantism to our model 4 from the Main Study. In this model we found that Purity still significantly positively effect on the belief that excess wealth was immoral ($\beta = .072, p = .002$) while Protestantism did not have a significant direct effect and did not influence this belief on its own (See Table S6).

To test whether the relationship between purity and beliefs about excessive wealth is moderated by country-level Protestantism, we next ran the same model adding the interaction between purity and Protestantism. Similar to our earlier models, Purity continues to have a significant positive effect ($\beta = .08, p < .001$) and Protestantism continues to not have a significant direct effect ($\beta = -.04, p = 0.778$). However, the Purity*Protestantism interaction had a significant negative effect ($\beta = -0.05, p = .003$), supporting the hypothesis that the effect of purity on the moral judgment of excessive wealth is significantly weaker in countries with higher levels of Protestantism (See Table S6).

Finally, to account for country-specific variations, we ran our interaction model with a random slope for purity, allowing the slope of purity to vary across countries. Similar to the previous models, purity had a significant positive effect ($\beta = 0.08, p = 0.017$), while Protestantism continued to have no significant direct effect ($\beta = -0.04, p = 0.795$). However, in this model, the purity X Protestantism interaction was no longer significant ($\beta = -0.04, p = 0.272$). When considering random slopes for purity, the interaction effect between purity and Protestantism loses significance, indicating potential variability in the moderation effect across countries.

The findings from these models provide detailed insights into how moral values and religious contexts interact to shape beliefs about wealth. Model 1 confirms that individuals who strongly endorse the moral foundation of purity are more likely to view excessive wealth as immoral. Model 2 further reveals that this relationship is moderated by country-level Protestantism, where higher levels of Protestantism are associated with a weaker link between purity and the belief that excessive wealth is immoral. This supports the idea that Protestant cultural contexts, which have

[1] https://en.wikipedia.org/wiki/Protestantism_by_country

Table S6 Multi-level Gaussian Models Predicting the Immorality of Excessive Wealth Including Country-level Protestantism

	Model 1	Model 2
(Intercept)	0.02	0.02
	(0.05)	(0.05)
Age	0.07^{***}	0.07^{***}
	(0.02)	(0.02)
Gender	0.01	0.01
	(0.02)	(0.02)
Religiosity	0.02	0.02
	(0.02)	(0.02)
Political Orientation	-0.05^{**}	-0.05^{**}
	(0.02)	(0.02)
Education	0.01	0.01
	(0.02)	(0.02)
Status	0.04^{*}	0.04^{*}
	(0.02)	(0.02)
Care	−0.02	−0.02
	(0.02)	(0.02)
Equality	0.33^{***}	0.33^{***}
	(0.02)	(0.02)
Proportionality	-0.08^{***}	-0.09^{***}
	(0.02)	(0.02)
Loyalty	-0.09^{***}	-0.09^{***}
	(0.02)	(0.02)
Authority	-0.11^{***}	-0.11^{***}
	(0.03)	(0.03)
Purity	0.07^{**}	0.08^{***}
	(0.02)	(0.02)
Moralization of Inequality	0.08^{***}	0.08^{***}
	(0.02)	(0.02)
Gini	−0.05	−0.05
	(0.06)	(0.06)
GDP	0.13^{*}	0.13^{*}
	(0.06)	(0.06)
Protestantism	−0.04	−0.04
	(0.06)	(0.06)
Purity × Protestantism		-0.05^{**}
		(0.02)
AIC	10830.57	10823.83
Log Likelihood	−5396.28	−5391.91
Num. obs.	4096	4096
Num. groups: country	20	20

$p < 0.001$; $^{**}p < 0.01$; $p < 0.05$

Note: Values outside parentheses represent the coefficient estimate and the values inside parentheses represent the standard error. All predictor variables were centered at the country-level.

historically been more accepting of wealth accumulation, can mitigate the moral condemnation of excessive wealth driven by purity concerns. In other words, generally in a country with high Protestantism (USA, South Africa, Kenya), the condemnation of excessive wealth has less to do with purity concerns than in less Protestant countries (Ireland, UAE, Belgium, Russia) where the condemnation of excessive wealth may have more to do with purity.

Fig. S3 *Plot of Interaction of Protestantism Moderating the Relationship Between Purity and the Moral Judgement of Excessive Wealth*

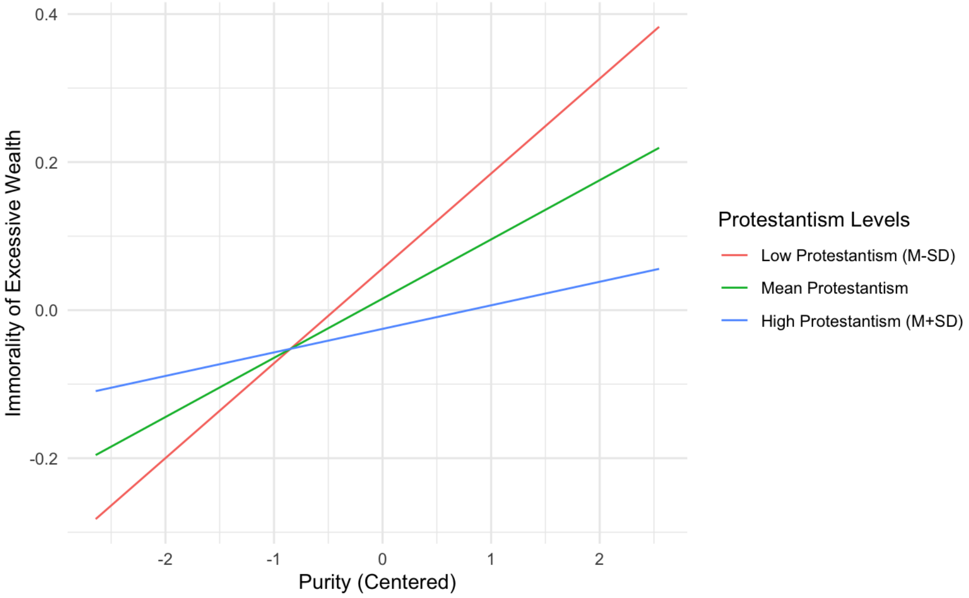

Note: Low Protestantism is one SD below the mean and High Protestantism is one SD above the mean

1.1.5 Additional Models with Country-level Interactions with Moral Foundations

In addition to the model above with country-level Protestantism interacting with the moral foundations, we ran two additional models that explored how our other two country-level variables, Gini and GDP, interact with the moral foundations. The results from the first model show that in countries with higher income inequality, the positive relationships that equality and purity have with the immorality of excessive wealth is weakened, evidenced by Gini's significant negative interaction with equality ($\beta = -0.057, p = .001$) and purity ($\beta = -0.084, p < .001$)(See Table S7). The results from the second model show a similar relationship, with GDP's significant negative interaction with purity ($\beta = -0.036, p = .044$) (See Table S8. The results from the third model are in agreement and reflect the earlier models on Protestantism, showing a significant negative interaction with urity ($\beta = -0.05, p = .041$) (See Table S9). Together, these results highlight that the positive relationship between purity and the belief in the immorality of excessive wealth is significantly less strong when in rich, unequal, and highly Protestant countries, suggesting that people find excessive wealth less of an impurity issue when excessive wealth is prevalent in their societies. These findings underscore the complexity of moral beliefs, demonstrating that both individual values and broader socio-economic and cultural contexts play crucial roles in determining feelings about too much money.

Table S7 Multi-level Gaussian Model Predicting the Immorality of Excessive Wealth Including Interactions Between Gini and Moral Foundations

	Model
(Intercept)	0.008
	(0.056)
Age	0.069^{***}
	(0.016)
Gender	0.011
	(0.015)
Religiosity	0.018
	(0.018)
Political Orientation	-0.050^{**}
	(0.016)
Education	0.008
	(0.015)
Status	0.037^{*}
	(0.016)
Care	−0.022
	(0.019)
Equality	0.330^{***}
	(0.017)
Proportionality	-0.082^{***}
	(0.019)
Loyalty	-0.092^{***}
	(0.024)
Authority	-0.116^{***}
	(0.025)
Purity	0.081^{***}
	(0.023)
Gini	$-0.118^{\cdot}$
	(0.065)
Moralization of Inequality	0.075^{***}
	(0.015)
GDP	0.016
	(0.077)
Protestantism	−0.019
	(0.069)
Care × Gini	$0.039^{\cdot}$
	(0.020)
Equality × Gini	-0.058^{**}
	(0.018)
Proportionality × Gini	−0.028
	(0.019)
Loyalty × Gini	−0.008
	(0.023)
Authority × Gini	$0.049^{\cdot}$
	(0.025)
Purity × Gini	-0.084^{***}
	(0.022)
AIC	10809.7
BIC	10967.6
Log Likelihood	−5379.8
Deviance	10759.7
Num. obs.	4096
Num. groups: country	20

$^{***}p < 0.001$; $^{**}p < 0.01$; $^{*}p < 0.05$; $^{\cdot}p < 0.1$

Note: Values outside parentheses represent the coefficient estimate and the values inside parentheses represent the standard error. All predictor variables were centered at the country-level.

Table S8 Multi-level Gaussian Model Predicting the Immorality of Excessive Wealth Including Interactions Between GDP and Moral Foundations

	Model
(Intercept)	0.009
	(0.056)
Age	0.070^{***}
	(0.016)
Gender	0.013
	(0.016)
Religiosity	0.019
	(0.018)
Political Orientation	-0.050^{**}
	(0.016)
Education	0.006
	(0.015)
Status	0.040^{**}
	(0.016)
Care	-0.029
	(0.020)
Equality	0.322^{***}
	(0.017)
Proportionality	-0.085^{***}
	(0.020)
Loyalty	-0.089^{***}
	(0.025)
Authority	-0.106^{***}
	(0.026)
Purity	0.086^{***}
	(0.023)
GDP	0.015
	(0.077)
Moralization of Inequality	0.077^{***}
	(0.015)
Gini	$-0.116^{\cdot}$
	(0.065)
Protestantism	-0.019
	(0.069)
Care × GDP	0.053^{**}
	(0.016)
Equality × GDP	0.005
	(0.015)
Proportionality × GDP	$0.029^{\cdot}$
	(0.016)
Loyalty × GDP	-0.016
	(0.022)
Authority × GDP	-0.024
	(0.022)
Purity × GDP	-0.036^{*}
	(0.018)
AIC	10824
BIC	10982
Log Likelihood	-5387
Deviance	10774
Num. obs.	4096
Num. groups: country	20

$^{***}p < 0.001$; $^{**}p < 0.01$; $^{*}p < 0.05$; $^{\cdot}p < 0.1$

Note: Values outside parentheses represent the coefficient estimate and the values inside parentheses represent the standard error. All predictor variables were centered at the country-level.

Table S9 Multi-level Gaussian Model Predicting the Immorality of Excessive Wealth Including Interactions Between Protestantism and Moral Foundations

	Model
(Intercept)	0.02
	(0.05)
Age	0.07^{***}
	(0.02)
Gender	0.01
	(0.02)
Religiosity	0.02
	(0.02)
Political Orientation	-0.05^{**}
	(0.02)
Education	0.01
	(0.02)
Status	0.04^{*}
	(0.02)
Care	−0.02
	(0.02)
Equality	0.33^{***}
	(0.02)
Proportionality	-0.09^{***}
	(0.02)
Loyalty	-0.09^{***}
	(0.02)
Authority	-0.11^{***}
	(0.03)
Purity	0.08^{***}
	(0.02)
GDP	0.13^{*}
	(0.06)
Moralization of Inequality	0.08^{***}
	(0.02)
Gini	−0.05
	(0.06)
Protestantism	−0.04
	(0.06)
Care × Protestantism	0.03
	(0.02)
Equality × Protestantism	−0.00
	(0.02)
Proportionality × Protestantism	0.01
	(0.02)
Loyalty × Protestantism	−0.01
	(0.02)
Authority × Protestantism	−0.00
	(0.03)
Purity × Protestantism	-0.05^{*}
	(0.02)
AIC	10831.91
Log Likelihood	−5390.95
Num. obs.	4096
Num. groups: country	20

$^{***}p < 0.001$; $^{**}p < 0.01$; $^{*}p < 0.05$; $^{\cdot}p < 0.1$

Note: Values outside parentheses represent the coefficient estimate and the values inside parentheses represent the standard error. All predictor variables were centered at the country-level.

—

1.1.6 The Gini Coefficient and the Moralization of Inequality

In Figure S4 below, the plot shows the relationship between our main country-level factor, the Gini coefficient, and our control variable, the moralization of inequality. This relationship was not significant (r_τ = -0.03, p = .871). This is the opposite relationship the Gini coefficient has with the immorality of excessive wealth which showed that the higher the inequality, the less immorality of excessive wealth. This suggests that moral feelings about inequality are not cleanly related to moral feelings of excessive wealth at the individual and national levels. Further research should explore this variation between the moralization of inequality and the immorality of excessive wealth across cultures since they appear to have a small but significant correlation (r = .11, $p < 0.001$).

Fig. S4 *Plot of Gini Coefficient and Moralization of Inequality by Country*

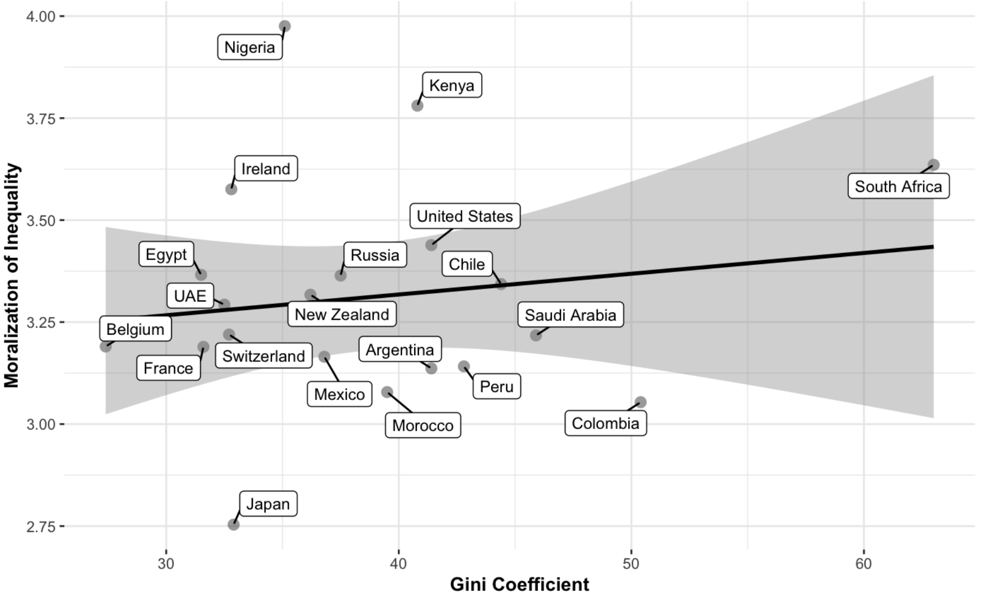

Note: Gini coefficient is scored from 0-100 where a country with perfect equality in which everyone earns and owns the same amount of wealth has a Gini coefficient of zero while a country with perfect inequality in which one person owns and earns everything would have a Gini coefficient of 100.

1.1.7 Dealing with Cross-National Non-Independence

In our initial country-level analysis, we find that the country-level Gini index of inequality was significantly associated with country-level immorality of excessive wealth (r_τ = -0.43, p = .007). However, because countries are connected in various ways (e.g. spatial proximity and shared cultural ancestry), many cross-national analyses may violate the assumptions of non-independence needed for such analysis, thus inflating the rates of false positives. In order to combat this issue, [6] recommend methods with additional controls to account for the non-independence of nations. Here, we included two of these methods, controlling first for spatial similarity between nations and then for cultural similarity via linguistic proximity.

Spatial Non-independence

We followed the steps as outlined by [6]. First, we fit a naive Bayesian regression model predicting the immorality of excessive wealth with the Gini coefficient, with set regularising priors on the intercept, slope, and residual variance. The effect of the Gini coefficient was found to be significantly negative, with an estimate of -0.02 (95% CI [-0.03, -0.00]), suggesting a slight decrease in the immorality of excessive wealth with an increase in inequality. These results show a significant relation between the two factors, similar to our results of the Kendell correlation reported in the main sections of the paper.

Next, in order to account for spatial non-independence, we fit another model, this time including a Gaussian Process (GP) over latitudes and longitudes for nations. This technique adds a random intercept for each nation, and these random intercepts are allowed to covary according to the distance between the coordinates [2]. This function processes variables to internally generate a normalized distance matrix for each case. It subsequently calculates a covariance function, which delineates the relationship between these distances and the covariance among nations. If there is spatial autocorrelation, the model will estimate strong spatial covariance between nations, which could "soak up" much of the relationship we saw in the previous section.

This indeed soaked up our relationship, as the Gini coefficient was now found to be insignificantly associated with MOEW, with an estimate of -0.00 (95% CI, [-0.02, 0.01]. This suggests, generally, that the relationship between inequality and the immorality of excessive wealth may have to do with factors related to geographic proximity. Consequently, a deeper question arises for further research: Does cross-cultural difference play a role when accounting for spatial auto-correlation?

Cultural Phylogenetic Non-independence via Linguistic Proximity

In addition to spatial similarity, it is also possible for nations to be culturally related and thus non-independent. For example, Spain and Mexico are halfway around the world from each other but are culturally similar in many respects due to the influence of Spanish colonialism. We thus should control for how countries are similar in culture.

As opposed to spatial similarity, cultural similarity does not have a fixed coordinate system like longitude and latitude, proposing an issue for Gaussian Processes

[2]See [16] for more details on this method of dealing with spatial autocorrelation

using the above method. Instead, [6] suggests another technique where we specify the covariance matrix in advance rather than estimate it in the model. In the case of cultural similarity, they suggest using the linguistic proximity between nations, weighted by the proportion of speakers of each language in each nation. Linguistic proximity expresses how closely related two languages are in the phylogeny of languages, which is then averaged over all languages spoken in each nation, weighted by the speaker percentages. To get a sense of the linguistic and cultural similarity, we plotted these scores in a correlation matrix below (See Figure S5). From this plot, we can see that the majority of Spanish-speaking nations have high linguistic proximity (i.e., cultural similarity), as do English-speaking nations and Arabic-speaking nations.

Fig. S5 *Plot of Cultural Similarity via Linguistic Proximity Across 20 Nations*

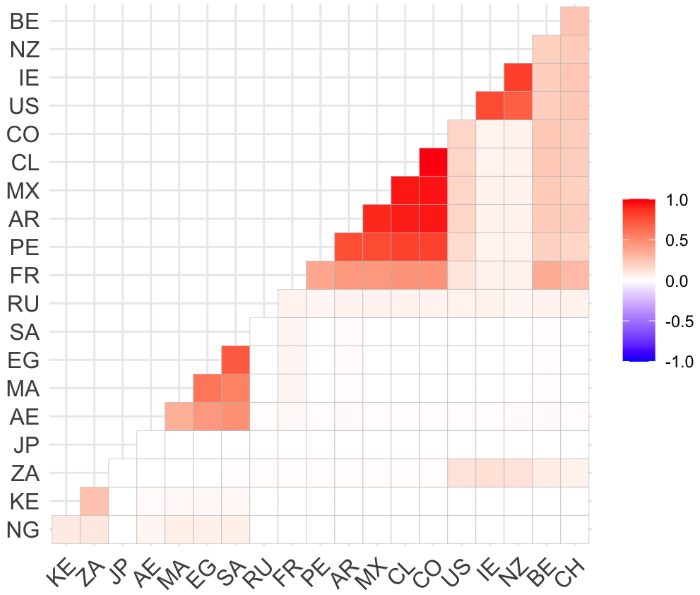

Note: BE = Belgium, NZ = New Zealand, IE = Ireland, US = United States, CO = Colombia, CL = Chile, MX = Mexico, AR = Argentina, PE = Peru, FR = France, RU = Russia, SA = Saudi Arabia, EG = Egypt, MA = Morocco, AE = United Arab Emirates, JP = Japan, ZA = South Africa, KE = Kenya, NG = Nigeria

We then used this linguistic proximity matrix in our modeling by specifying in advance that this is how our random effects should be correlated. Once again, as expected, this results in soaking up our significant association between Gini and

immorality of excessive wealth, with an estimate of -0.01 (95% CI, [-0.03, 0.01]. This suggests that the association between country-level inequality and the immorality of excessive wealth may be related to cultural-linguistic similarities, similar to the results of spatial similarity.

1.2 Country-level Corruption and the Immorality of Excessive Wealth

While our main hypotheses concerned how country-level economic inequality (i.e., the Gini coefficient) may influence the immorality of excessive wealth, country-level corruption may also influence the moralization of owning too much money. Instead of the mere presence of people who have a disproportionate amount of wealth, perhaps the ethics surrounding how the wealth was gained and how the wealth is spent determines whether people believe having excessive wealth is wrong or not. It may not be wealth that corrupts (the soul of individuals), but rather corrupt practices (e.g., nepotism) result in the extreme wealth of a few. This analysis was conducted as suggested by a reviewer, but we did not predict this relationship because while "corruption" at the country level has the same name as "corruption" of individuals' souls, it is more related to moral parochialism and nepotism, which have been shown to be related to loyalty (and perhaps authority) rather than the moral foundation of purity. At the individual level of analysis, purity dictates staying away from things and practices that can stain, corrupt, or degrade the sanctity of the human mind and/or body. Money, excessive amounts of it beyond what can be used to have a decent life, can be perceived as a means that permits people to do whatever they want, making them less cooperative and hence less moral. To rule out the idea that country-level corruption shapes the immorality of excessive wealth, we ran the following analyses.

1.2.1 Method

Measure

We used the Global Corruption Index (GCI) [7] as our measure for country-level corruption. GCI aggregates the results of multiple subcategories, including Corruption Perception, Corruption Experience, Citizen Voice and Transparency, Government Functioning and Effectiveness, Legal Context, Political Context, and White Collar Crimes.[3] GCI is scored on a scale of 0-100, where 0 represents the lowest risk of corruption and white collar crimes, and 100 corresponds to the highest risk of corruption and white collar crimes.

Analytic Procedure

We ran the same analytic procedure with GCI measures for each of our 20 countries as the main paper. To examine the relationship between moral values and the immorality of excessive wealth, we employed multilevel models to account for the clustered nature of our data. Our individual-level variables included self-report measures

[3] We chose this measure over the widely used Corruption Perception Index (CPI) because the latter does not measure white collar crimes, activities such as tax fraud, money laundering, financial secrecy or illicit flows of money, which we believe is directly related to our idea of the morality of excessive wealth.

(care, equality, proportionality, loyalty, authority, purity, moralization of inequality, immorality of excessive wealth) and demographic variables (age, gender, subjective SES, political conservatism, religiosity, and education). Our country-level variables were the GCI ratings of corruption, the Gini index of inequality, GDP per capita, and the percentage of Protestantism. We employed five multi-level models with random intercepts and no random slopes, consecutively adding more control variables to the base model. We used the “lme4” package, version 4.0.1, in R programming language for statistical analysis.[4]

1.2.2 Results

Country-level Analysis

The GCI was unrelated to the immorality of excessive wealth (r_τ = -0.23, p = .153; see Figure S6) and unrelated to moralization of inequality (r_τ = 0.12, p = .455).

Fig. S6 *The Relationship Between the Global Corruption Index (GCI) and Immorality of Excessive Wealth*

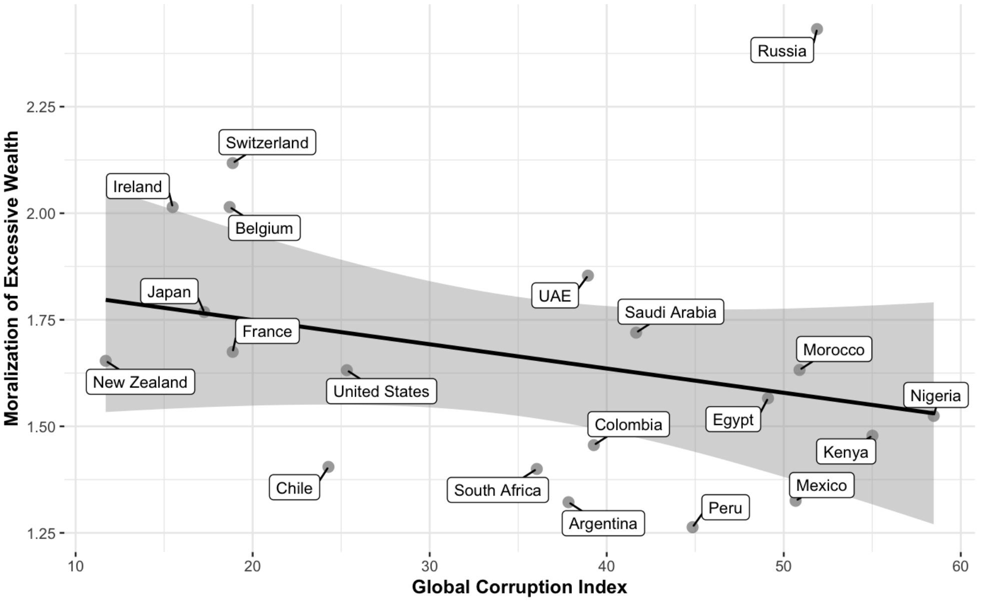

Note: GCI is scored on a scale of 0-100, where 0 represents the lowest risk of corruption and white-collar crimes, and 100 corresponds to the highest risk of corruption and white-collar crimes.

[4]https://cran.r-project.org/web/packages/lme4/index.html

Fig. S7 *The Relationship Between the Global Corruption Index (GCI) and Moralization of Inequality*

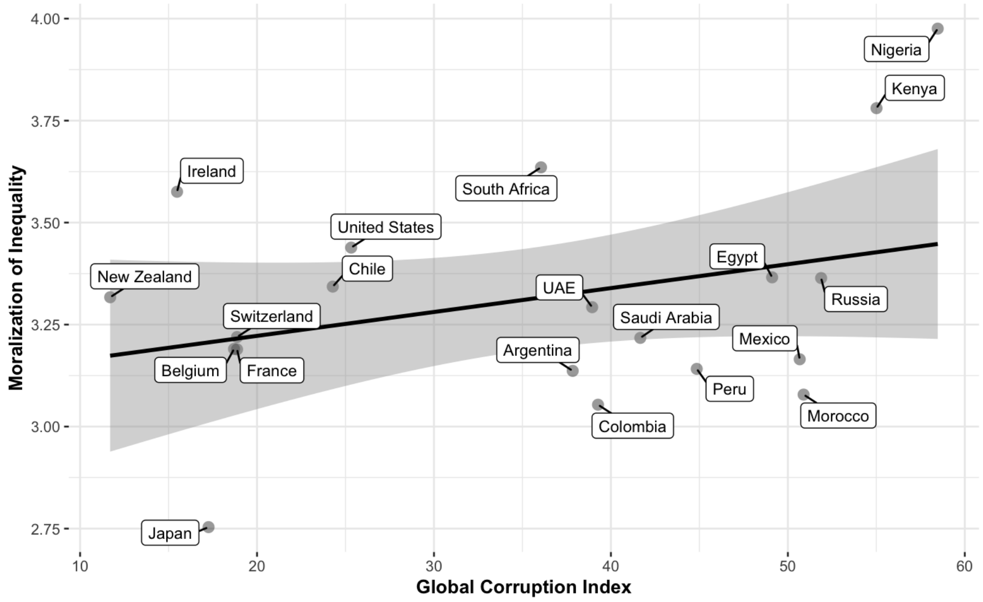

Note: GCI is scored on a scale of 0-100, where 0 represents the lowest risk of corruption and white-collar crimes, and 100 corresponds to the highest risk of corruption and white-collar crimes.

Multi-level Analysis

Overall, when including corruption, our final model's results remain similar in patterns of relationships. For individual level variables, equality($\beta = 0.32, p < .001$), purity($\beta = 0.08, p < .001$), moralization of inequality ($\beta = 0.07, p < .001$), status ($\beta = 0.04, p = .041$), and age ($\beta = 0.08, p < .001$) positively predict immorality of excessive wealth significantly while proportionality ($\beta = -0.08, p < .001$), loyalty ($\beta = -0.08, p < .001$), authority ($\beta = -0.10, p < .001$), and conservatism ($\beta = -0.05, p = .006$) are negatively associated. For country-level factors, only GDP per capita was significant ($\beta = 0.20, p = .041$) There were also some significant interaction terms between the Gini coefficient and both care ($\beta = -0.05, p = .01$) and purity ($\beta = -0.08, p < .001$) (See Table S10). As can be seen, country-level corruption does not predict the immorality of excessive wealth. Further, it does not add any predictive value in the multi-level linear models (Compare model 4 in Table S10 to model 4 in the Table in the main text).

Table S10 Multi-level Gaussian Models Predicting the Immorality of Excessive Wealth with Corruption and Inequality as Country-level Variables

	Model 1	Model 2	Model 3	Model 4	Model 5
(Intercept)	0.00	0.01	0.02	0.02	0.02
Care	−0.03	−0.02	−0.02	−0.02	−0.03
Equality	0.33***	0.33***	0.33***	0.33***	0.32***
Proportionality	−0.09***	−0.08***	−0.08***	−0.08***	−0.08***
Loyalty	−0.08**	−0.09***	−0.09***	−0.09***	−0.08***
Authority	−0.12***	−0.11***	−0.11***	−0.12***	−0.10***
Purity	0.08***	0.07**	0.07**	0.08***	0.08***
Moralization of Inequality	0.08***	0.08***	0.08***	0.08***	0.07***
Age		0.07***	0.07***	0.07***	0.08***
Gender		0.01	0.01	0.01	0.01
Religiosity		0.02	0.02	0.02	0.02
Conservatism		−0.05**	−0.05**	−0.05**	−0.05**
Education		0.01	0.01	0.01	0.01
Status		0.04*	0.04*	0.04*	0.04*
Gini			−0.04	−0.04	−0.04
GDP Per Capita			0.20*	0.20*	0.20*
Protestantism			−0.05	−0.05	−0.05
GCI			0.08	0.09	0.08
Care X GCI				−0.03	−0.04*
Equality X GCI				−0.05**	−0.04*
Proportionality X GCI				−0.02	−0.02
Loyalty X GCI				−0.00	0.00
Authority X GCI				0.05*	0.04
Purity X GCI				−0.03	−0.01
Care X Gini					0.05*
Equality X Gini					−0.05**
Proportionality X Gini					−0.02
Loyalty X Gini					−0.01
Authority X Gini					0.04
Purity X Gini					−0.08***
AIC	11481.54	10834.31	10831.14	10805.47	10791.97
Log Likelihood	−5730.77	−5401.16	−5395.57	−5376.74	−5363.99
Num. obs.	4342	4096	4096	4096	4096
Num. groups: country	20	20	20	20	20

$^{***}p < 0.001$; $^{**}p < 0.01$; $^{*}p < 0.05$

Note: Numbers represent the coefficient estimate and asterisks represent significance.

1.3 Morality of Excess Surveys

In terms of our main findings, it may be the moral attitudes about how people spend and acquire their wealth that underlie the morality of excessive wealth. For example, people high in purity might find excessive wealth as morally wrong because they assume that person acquired that wealth in an exploitative way or spends that money in a selfish way. Additionally, purity may underlie the morality of excess, regardless of whether that is excessive wealth or excess in other domains. In order to further clarify the morality of excessive wealth, we explore three lingering questions: 1) How do people feel about the morality of how excessive wealth is gained? 2) How do people feel about the morality of how excessively wealthy people spend their wealth? and 3) How do people feel about the morality of excess in domains other than wealth?

1.4 Pilot Study

We first ran a pilot study to explore the various ways that people moralize wealth acquisition, wealth spending, and excess beyond wealth cluster into any general categories. We also aimed to do some preliminary psychometric tests to finalize the materials for the pre-registered Supplementary Study.

1.4.1 Method

Participants

For our pilot study, we gathered 100 participants from Prolific platforms, balanced on gender (50% female).

Measures

The MFQ-2, morality of excessive wealth, political ideology, religiosity, and age were all measured in the same manner as the study in the main text. In order to measure the morality of excess beyond wealth, we gave 26 questions which asked "Is it morally wrong to have too much [ITEM]?" [Knowledge, Competence, Equality, Happiness, Mom Love, Ambition, Power, Patriarchy, Competition, Loyalty, Authority, Work, Environmental footprint, prayer, Health, Wisdom, Friends, Hair, Sex, Eat, Arrogance, Fun, Anger].

In order to measure the moral attitudes of excessive wealth spending, we asked "Below you will see a number of different explanations about how extremely rich people gained an excessive amount of wealth. Please rate how moral you find these different paths to wealth acquisition on a scale of (1) not wrong at all to (5) Extremely wrong. " with the following items: Philanthropy, Innovative Impact, Entrepreneurship, Arts and Culture, Healthcare and Medical Research, Lavish Lifestyle, Lobbying, Tax Evasion, Corruption, and Wealth Management.

In order to measure the moral attitudes of wealth acquisition, we asked "Below you will see a number of different explanations about how extremely rich people gained an excessive amount of wealth. Please rate how moral you find these different paths to wealth acquisition on a scale of 1) not wrong at all to (5) Extremely wrong" with the following items: Entertainment, Inheritance, Genius, Luck, Athletics, Fair Production, Government corruption, Corporate corruption, Nepotism, and Worker Exploitation.

Analytic Procedure

We ran exploratory factor analysis using parallel analysis for our three groups of questions (Morality of Excess, Morality of Spending Excessive Wealth, and Morality of Excessive Wealth Acquisition) to assess whether certain items load together. Our minimum threshold for Cronbach's Alpha was .60.

1.4.2 Pilot Results EFA

Morality of Excess

Results from EFA using parallel analysis broke down our set of items into four main factors (Benevolent Excess, Exploitative Excess, Constructive Excess, and Self Indulgence Excess). Since some of these scales have few items, our minimum threshold for Cronbach's alpha is 0.60. The items for Benevolent Excess include Knowledge, Competence, Equality, Happiness, Mom Love, and Ambition. The items for Exploitative Excess include Power, Patriarchy, Competition, Loyalty, Authority, Work, Environmental footprint, and prayer. The items for Constructive Excess include Health, Wisdom, Friends, and hair. The items for Self-Indulgence Excess include Sex, Eating, Arrogance, Fun, and Anger.

The Exploratory Factor Analysis (EFA) with parallel analysis and varimax rotation, conducted to assess the factor structure within the dataset of items related to the moralization of spending excessive wealth, resulted in a four-factor solution. The Kaiser-Meyer-Olkin (KMO) measure of sampling adequacy was robust at 0.83, indicating that the dataset was suitable for factor analysis. Bartlett's test for sphericity was highly significant ($\chi^2 = 1378.18, df = 276, p < .001$), confirming that the variables were intercorrelated and suitable for EFA. The analysis revealed that the four factors explained a cumulative variance of 54%, with individual contributions of 16%, 15%, 13%, and 11% for each factor, respectively. Factor loadings suggested diverse contributions across items, with notable high loadings across multiple factors, demonstrating the multidimensionality of moral considerations in the context of excess. The Tucker-Lewis Index of factoring reliability stood at 0.826, and the RMSEA index was 0.082, within acceptable limits, suggesting a good fit for the model. This four-factor model elucidates complex moral frameworks individuals might use when considering excess in various domains. Given this split, we labeled the four factors as Benevolent Excess, Exploitative Excess, Constructive Excess, and Self Indulgence Excess (See Table Factor Loadings and Variance Explained in EFA on Items Related to the Morality of Spending Excessive Wealth.

Morality of Spending Excessive Wealth

The Exploratory Factor Analysis (EFA) with parallel analysis and varimax rotation, conducted to assess the factor structure within the dataset of items related to the moralization of spending excessive wealth, resulted in a two-factor solution. The Kaiser-Meyer-Olkin (KMO) test yielded an overall measure of sampling adequacy (MSA) of 0.88, indicating that the data was well-suited for factor analysis. Individual item MSAs ranged from 0.60 to 0.95, with most items showing high adequacy.

Table S11 Factor Loadings and Variance Explained in EFA on Items Related to the Morality of Excess

Item	Exploitative	Constructive	Self-Indulgence	Benevolent
Knowledge	0.143	0.311	.	0.474
Health	.	0.753	.	0.341
Competence	0.239	0.233	.	0.756
Loyalty	0.511	0.114	0.155	0.342
Authority	0.740	.	0.238	0.208
Equality	.	0.371	.	0.494
Wisdom	0.118	0.826	.	0.201
Friends	0.189	0.801	.	0.239
Hair	.	0.801	0.130	0.176
Sex	.	0.168	0.430	0.403
Happiness	0.234	0.173	0.112	0.761
Work	0.479	0.215	0.390	0.116
Eat	0.255	0.104	0.535	0.207
Environment	0.533	0.117	.	.
Prayer	0.546	0.381	0.109	0.192
Mom Love	0.393	0.343	.	0.423
Power	0.734	.	0.302	0.144
Patriarchy	0.786	.	0.195	0.165
Arrogance	0.317	-0.107	0.598	.
Fun	0.181	0.199	0.513	0.471
Competition	0.432	.	0.349	0.406
Ambition	0.339	0.365	0.323	0.449
Anger	0.202	.	0.844	.
Water	0.308	0.288	0.339	.
SS loadings	3.751	3.506	2.573	3.100
Proportion Var	0.156	0.146	0.107	0.129
Cumulative Var	0.156	0.302	0.432	0.539

Bartlett's test for sphericity was significant ($\chi^2 = 562.53, df = 36, p < .001$), supporting the factorability of the correlation matrix. The scree plot and parallel analysis suggested a two-factor solution. In this EFA using the minimum residual method with varimax rotation, the two extracted factors explained 65% of the total variance, with the first factor (MR1) accounting for 48% and the second factor (MR2) for 17%. The factor loadings for items like Philanthropy, Impact, Entrepreneurship, Art, and Health were notably high on MR1, whereas Lobby, Tax Evasion, and Corruption loaded significantly on MR2. The root mean square of the residuals (RMSR) was very low at 0.03, and the Tucker-Lewis Index of factoring reliability was excellent at 1.003. The RMSEA index was 0, indicating a perfect model fit. These results underscore the robustness and adequacy of the two-factor model in capturing the underlying dimensions of moralization of spending excessive wealth in the dataset. Given this split, we labeled the two factors as benevolent spending and exploitative spending (See Table Factor Loadings and Variance Explained in EFA on Items Related to the Morality of Spending Excessive Wealth.

Morality of Acquiring Excessive Wealth

The Exploratory Factor Analysis (EFA) with parallel analysis and varimax rotation, conducted to assess the factor structure within the dataset of items related to the

Table S12 Factor Loadings and Variance Explained in EFA on Items Related to the Morality of Spending Excessive Wealth

Item	Benevolent Spending	Exploitative Spending
Philanthropy	0.870	
Impact	0.821	
Entrepreneurship	0.921	
Art	0.859	0.141
Health	0.851	
Lobby	0.114	0.568
Tax Evasion	0.114	0.688
Corruption	-0.102	0.826
Wealth Management	0.732	0.215
SS loadings	4.313	1.553
Proportion Var	0.479	0.173
Cumulative Var	0.479	0.652

immorality of excessive wealth acquisition, resulted in a two-factor solution. The analysis, performed using the minimum residual method (*minres*), revealed substantial factor loadings on two dimensions, designated as MR1 and MR2. The total variance explained by these factors was 59%, with MR1 accounting for a larger proportion (40%) compared to MR2 (18%). The proportion of variance explained by MR1 and MR2 was 69% and 31%, respectively. The mean item complexity was 1.1. The root mean square of the residuals (RMSR) was low at 0.04, indicating a good fit. The Tucker-Lewis Index of factoring reliability was high (0.972), and the RMSEA index was 0.053, falling within acceptable limits. The factor loadings for specific variables like Inheritance, Entertainment, Athletics, Genius, and Luck were notably high on MR1, whereas Corporate Corruption, Government Corruption, and Nepotism loaded significantly on MR2. This two-factor model demonstrated good statistical and practical significance, elucidating the underlying structure of the dataset. Given this split, we labeled the two factors as benevolent acquisition and exploitative acquisition (See Table Factor Loadings and Variance Explained in EFA on Items Related to the Morality of Acquiring Excessive Wealth.

Table S13 Factor Loadings and Variance Explained in EFA on Items Related to the Morality of Acquiring Excessive Wealth

Item	Benevolent Acquisition	Exploitative Acquisition
Inheritance	0.843	
Exploitation		0.460
Corporate Corruption	-0.174	0.785
Government Corruption	-0.176	0.803
Benevolence	0.657	
Entertainment	0.857	
Athletics	0.807	
Genius	0.829	-0.109
Luck	0.821	
Nepotism	0.300	0.564
SS loadings	4.043	1.813
Proportion Var	0.404	0.181
Cumulative Var	0.404	0.586

1.5 Main Study

Given the results of our pilot, we then ran a fully powered study that measured how core moral values, moral values about excessive wealth acquisition, and moral values about excessive wealth spending predict the immorality of excessive wealth and other types of excess.

1.5.1 Methods

Participants

For our main study, we gathered 330 participants from Prolific platforms, balanced on gender (50% female) and political orientation (50% conservative). We conducted a power analysis in R programming using the “pwr” package and pwr.f2.test function to calculate the sample size for our regression analyses. For parameters we used $f2 = 0.1$ (for a small to medium effect size), p = 13 (for our total number of predictors including our eight main [Care, Equality, Proportionality, Loyalty, Authority, Purity, Religion, Politics] and 5 controls [Age, Benevolent Spending, Exploitative Spending, Benevolent Acquisition, Exploitative Acquisition], significance level = 0.05 (significance level), power = 0.95 (our desired power). This resulted in a suggested sample size of 277. To ensure we will have enough power after excluding participants who fail attention checks or leave parts of the survey blank, we will aim to recruit 330 participants.

Measures

Mean scores were calculated for questions related to each of the following factors listed above in our exploratory factor analysis: Benevolent Spending, Exploitative Spending, Benevolent Acquisition, Exploitative Acquisition, Benevolent Excess, Exploitative Excess, Constructive Excess, Self Indulgence Excess

Analytic Procedure

We used the same analytic procedure as the pilot study, running iterative regression models to test how core moral values, the morality of excessive wealth, the morality of excess beyond wealth, the morality of excessive spending, and the morality of wealth acquisition, controlling for gender and political ideology.

1.5.2 Results

In a series of multiple linear regression analyses predicting the immorality of excessive wealth, our results indicate significant changes in predictive strength and variable significance as our models incorporated additional variables (See table S14). Model 1, with a limited set of predictors, revealed significant effects for the endorsement of equality ($\beta = 0.22$, $p < .001$) and authority ($\beta = -0.38$, $p < .001$), suggesting initial perceptions were influenced by views on equality and authority. The introduction of variables related to spending habits in Model 2 significantly improved the model fit (Adj. R^2 from 0.28 to 0.48), with 'Benevolent Spending' (β = 0.74, p ¡ .001) and 'Exploitative Spending' (β = 0.20, p ¡ .001) emerging as strong predictors, indicating moral attitudes towards spending habits are strongly related to the immorality

of excessive wealth. Model 3 further refined these insights by adding variables on acquisition methods, leading to 'Benevolent Acquisition' being a significant predictor ($\beta = 0.39$, $p < .001$), while maintaining the significance of 'Benevolent Spending' and 'Exploitative Spending' though with reduced coefficients. Endorsement of authority remained consistently significant across all models, with diminished effect in the final model ($\beta = -0.21$, $p < .05$). The progression of these models underscores the nuanced role of individual values and economic behaviors in shaping moral judgments of wealth, with the final model achieving the highest explanatory power (Adj. $R^2 =$ 0.51).

Table S14 Results from Multiple Linear Regression Models Predicting the Immorality of Excessive Wealth

	Model 1	Model 2	Model 3
(Intercept)	3.08^{***}	1.17^{**}	0.73
Care	0.05	0.05	0.08
Equality	0.22^{***}	0.06	0.01
Proportionality	-0.20^{*}	-0.09	-0.11
Loyalty	0.07	-0.04	-0.02
Authority	-0.38^{***}	-0.21^{*}	-0.21^{*}
Purity	0.08	-0.00	-0.01
Age	-0.01^{**}	-0.01^{*}	-0.01^{*}
Conservatism	0.09	0.03	0.07
Religiosity	-0.01	-0.04	-0.04
Benevolent Spending		0.74^{***}	0.47^{***}
Exploitative Spending		0.20^{***}	0.17^{**}
Benevolent Acquisition			0.39^{***}
Exploitative Acquisition			0.09
R^2	0.30	0.50	0.53
Adj. R^2	0.28	0.48	0.51
Num. obs.	315	315	315

$^{***}p < 0.001$; $^{**}p < 0.01$; $^{*}p < 0.05$

The results from multiple linear regression models predicting the moralization of benevolent excess (See Table S15) illustrate a gradual enhancement in the models' explanatory power and the emergence of significant predictors as variables are added across models. Initially, Model 1 highlights a minimal set of predictors with purity ($\beta = 0.09$, $p < .01$) and care ($\beta = -0.06$, $p < .05$) showing significant relationships, although with low overall explanatory power (Adj. $R^2 = 0.05$). The introduction of spending habits in Model 2 substantially increases the model's explanatory power (Adj. $R^2 = 0.20$), with the moralization of benevolent spending emerging as a strong positive predictor of the moralization of benevolent excess ($\beta = 0.22$, $p < .001$). Model 3 slightly refines the predictive accuracy (Adj. $R^2 = 0.22$) and reveals benevolent acquisition as an additional significant predictor ($\beta = 0.12$, $p < .01$), while benevolent spending remains significant ($\beta = 0.13$, $p < .001$). The consistency of purity across all models underscores the moral valuation of purity in the context of benevolent excess and its relationship with excess in general.

Table S15 Results from Multiple Linear Regression Models Predicting the Moralization of Benevolent Excess

	Model 1	Model 2	Model 3
(Intercept)	1.35^{***}	1.03^{***}	0.92^{***}
Care	-0.06^{*}	-0.04	-0.03
Equality	0.01	-0.03	-0.04^{*}
Proportionality	-0.01	0.03	0.02
Loyalty	0.06	0.03	0.03
Authority	-0.05	-0.01	-0.01
Purity	0.09^{**}	0.06^{*}	0.06^{*}
Age	-0.00^{*}	-0.00	-0.00
Conservatism	0.05	0.04	0.06
Religiosity	-0.01	-0.01	-0.01
Benevolent Spending		0.22^{***}	0.13^{***}
Exploitative Spending		-0.03	-0.03
Benevolent Acquisition			0.12^{**}
Exploitative Acquisition			0.02
R^2	0.08	0.23	0.26
Adj. R^2	0.05	0.20	0.22
Num. obs.	315	315	315

$^{***}p < 0.001$; $^{**}p < 0.01$; $^{*}p < 0.05$

The regression models predicting the moralization of exploitative excess (See Table S16)illustrate a gradual enhancement in the models' explanatory power and the emergence of significant predictors as variables are added across models. The initial model identifies the endorsement of authority as a significant negative predictor ($\beta = -0.23$, p ¡ .01) and equality as a positive predictor ($\beta = 0.10$, p ¡ .05), indicating that views on authority and equality significantly impact moral perceptions of exploitative behavior. With the inclusion of spending habits in Model 2, the explanatory power significantly increases (Adj. $R^2 = 0.37$), and benevolent spending ($\beta = 0.45$, $p <$ .001), and exploitative spending' ($\beta = 0.12$, $p < .01$) emerge as strong positive predictors, suggesting that the moralization of spending behaviors are strongly related to the moral assessments of exploitative excess. In the final model (Model 3), the addition of acquisition methods further enhances the model's explanatory capacity (Adj. $R^2 = 0.42$) and introduces benevolent acquisition ($\beta = 0.21$, $p < .01$) and exploitative acquisition' ($\beta = 0.22$, $p < .001$) as significant predictors. Conservatism also becomes a significant predictor in Model 3 ($\beta = 0.19$, $p < .05$), highlighting the role of ideological beliefs in the moralization process of exploitative excess.

In the regression analyses that predict the moralization of constructive excess, Model 1 starts by establishing a baseline with significant effects for the endorsement of care ($\beta = -0.06$, p < .01), equality ($\beta = 0.04$, p < .05), loyalty ($\beta = 0.06$, p < .05), purity ($\beta = 0.07$, p < .01), and age ($\beta = -0.00$, p < .001), suggesting that moral values and age significantly predict moral judgments on constructive excess, however with a moderate model fit (Adj. $R^2 = 0.12$). The introduction of spending habits in Model 2 notably improves the model's fit (Adj. $R^2 = 0.22$), with the moralization of benevolent spending becoming a strong positive predictor ($\beta = 0.15$, p ¡ .001). Model 3 slightly increases the model's explanatory capacity (Adj. $R^2 = 0.24$) and introduces the moralization of benevolent acquisition as an additional significant variable ($\beta = 0.09$, p < .01). The consistent significance of Purity across all models and the sustained

Table S16 Results from Multiple Linear Regression Models Predicting the Moralization of Exploitative Excess

	Model 1	Model 2	Model 3
(Intercept)	2.21***	1.06***	0.46
Care	−0.03	−0.02	−0.03
Equality	0.10*	0.00	−0.02
Proportionality	0.01	0.08	0.04
Loyalty	−0.02	−0.08	−0.07
Authority	−0.23**	−0.12	−0.12
Purity	0.08	0.03	0.05
Age	−0.00	−0.00	−0.00
Conservatism	0.17	0.13	0.19*
Religiosity	−0.00	−0.02	−0.02
Benevolent Spending		0.45***	0.30***
Exploitative Spending		0.12**	0.03
Benevolent Acquisition			0.21**
Exploitative Acquisition			0.22***
R^2	0.21	0.39	0.44
Adj. R^2	0.19	0.37	0.42
Num. obs.	315	315	315

$^{***}p < 0.001$; $^{**}p < 0.01$; $^{*}p < 0.05$

impact of age on moralization once again emphasizes the role of purity and age in the moralization of excess in general.

Table S17 Results from Multiple Linear Regression Models Predicting the Moralization of Constructive Excess

	Model 1	Model 2	Model 3
(Intercept)	1.02***	0.84***	0.84***
Care	−0.06**	−0.05*	−0.04
Equality	0.04*	0.01	−0.00
Proportionality	0.01	0.04	0.04
Loyalty	0.06*	0.04	0.04
Authority	−0.02	0.01	0.00
Purity	0.07**	0.05*	0.04*
Age	−0.00***	−0.00***	−0.00***
Conservatism	0.06	0.06	0.06
Religiosity	−0.00	−0.01	−0.01
Benevolent Spending		0.15***	0.09**
Exploitative Spending		−0.03	−0.02
Benevolent Acquisition			0.09**
Exploitative Acquisition			−0.03
R^2	0.14	0.25	0.27
Adj. R^2	0.12	0.22	0.24
Num. obs.	315	315	315

$^{***}p < 0.001$; $^{**}p < 0.01$; $^{*}p < 0.05$

Lastly, the regression analyses for predicting the moralization of selfish-indulgence excess reveal significant predictors across three models, with the endorsement of purity and level of conservatism consistently emerging as key influences, emphasizing the importance of moral and ideological values in judgments of selfish indulgence

(See Table S18). The inclusion of the moralization of spending habits and acquisition behaviors in subsequent models significantly enhances the explanatory power, indicating that the attitudes towards financial behaviors also critically shape moral perceptions of excessive self-indulgence. Notably, the negative significance of the endorsement of care in later models underscores the complex relationship between compassion and judgments of indulgence. The stable strong association with purity is in close alignment with our main study's finding on purity's role in the immorality of excessive wealth.

Table S18 Results from Multiple Linear Regression Models Predicting the Moralization of Selfish-Indulgence Excess

	Model 1	Model 2	Model 3
(Intercept)	0.89*	−0.03	−0.71
Care	−0.09	−0.11*	−0.12*
Equality	0.06	−0.00	−0.03
Proportionality	0.04	0.06	0.02
Loyalty	0.01	−0.03	−0.01
Authority	0.05	0.12	0.12
Purity	0.40***	0.38***	0.40***
Age	−0.00	−0.00	−0.00
Conservatism	0.35***	0.31***	0.37***
Religiosity	0.00	−0.01	−0.02
Benevolent Spending		0.22***	0.07
Exploitative Spending		0.18***	0.08
Benevolent Acquisition			0.21**
Exploitative Acquisition			0.26***
R^2	0.25	0.33	0.38
Adj. R^2	0.23	0.30	0.35
Num. obs.	315	315	315

$^{***}p < 0.001$; $^{**}p < 0.01$; $^{*}p < 0.05$

1.6 Survey Questions Main Study

For the main study, the following questions were asked with both the block order randomized and the questions within each block were also randomized. All questions were asked in the following languages for each country: Argentina/Spanish, Belgium/French, Chile/Spanish, Colombia/Spanish, Egypt/Arabic, France/French, Ireland/English, Japan/Japanese, Kenya/English, Mexico/Spanish, Morocco/Arabic, New Zealand/English, Nigeria/English, Peru/Spanish, Russia/Russian, Saudi Arabia/Arabic, South Africa/English, Switzerland/French, United Arab Emirates/Arabic.

1.6.1 Demographics

1. What is your first language?
2. What is your Religious affiliation? [Christianity; Islam;Judaism;Hinduism;Buddhism;Nonreligious]
3. On a scale from 0-10, how religious do you consider yourself?

4. What is the highest educational level that you have attained?[Less than high school; High school graduate; Some college; Bachelor's degree; Master's degree; Professional degree or doctorate]
5. Think of a ladder with 10 steps representing where people stand in your country. At step 10 are people who are the best off – those who have the most money, the most education, and the most respected jobs. At step 1 are the people who are worst off – those who have the least money, least education, and the least respected jobs or no job. Where would you place yourself on this ladder?
6. People often describe their politics as left-wing (progressive) or right-wing (conservative). On a scale (1-7), where would you position yourself?

1.6.2 MFQ-2

Moral Foundations Questionnaire-2 [2] For each of the statements below, please indicate how well each statement describes you or your opinions. Response options: Does not describe me at all (1); Slightly describes me (2); Moderately describes me (3); Describes me fairly well (4); Describes me extremely well (5).

1. Caring for people who have suffered is an important virtue.
2. The world would be a better place if everyone made the same amount of money.
3. I think people who are more hard-working should end up with more money.
4. I think children should be taught to be loyal to their country.
5. I think it is important for societies to cherish their traditional values.
6. I think the human body should be treated like a temple, housing something sacred within.
7. I believe that compassion for those who are suffering is one of the most crucial virtues.
8. Our society would have fewer problems if people had the same income.
9. I think people should be rewarded in proportion to what they contribute.
10. It upsets me when people have no loyalty to their country.
11. I feel that most traditions serve a valuable function in keeping society orderly.
12. I believe chastity is an important virtue.
13. We should all care for people who are in emotional pain.
14. I believe that everyone should be given the same quantity of resources in life.
15. The effort a worker puts into a job ought to be reflected in the size of a raise they receive.
16. Everyone should love their own community.
17. I think obedience to parents is an important virtue.
18. It upsets me when people use foul language like it is nothing.
19. I am empathetic toward those people who have suffered in their lives.
20. I believe it would be ideal if everyone in society wound up with roughly the same amount of money.
21. It makes me happy when people are recognized on their merits.
22. Everyone should defend their country, if called upon.
23. We all need to learn from our elders.

24. If I found out that an acquaintance had an unusual but harmless sexual fetish I would feel uneasy about them.
25. Everyone should try to comfort people who are going through something hard.
26. When people work together toward a common goal, they should share the rewards equally, even if some worked harder on it.
27. In a fair society, those who work hard should live with higher standards of living.
28. Everyone should feel proud when a person in their community wins in an international competition.
29. I believe that one of the most important values to teach children is to have respect for authority.
30. People should try to use natural medicines rather than chemically identical human-made ones.
31. It pains me when I see someone ignoring the needs of another human being.
32. I get upset when some people have a lot more money than others in my country.
33. I feel good when I see cheaters get caught and punished.
34. I believe the strength of a sports team comes from the loyalty of its members to each other.
35. I think having a strong leader is good for society.
36. I admire people who keep their virginity until marriage.

Scoring: Average each of the following items to get six scores corresponding with the six foundations. Care = 1, 7, 13, 19, 25, 31 Equality = 2, 8, 14, 20, 26, 32 Proportionality = 3, 9, 15, 21, 27, 33 Loyalty = 4, 10, 16, 22, 28, 34 Authority = 5, 11, 17, 23, 29, 35 Purity = 6, 12, 18, 24, 30, 36

1.6.3 Wealth and Inequality

1. On a scale from 1 (*Not wrong at all*) to 5 (*Extremely wrong*), "Is it morally wrong to have too much money?".
2. One a scale from 1 (*Not at all*) to 5 (*Very much*), "How much are your feelings about inequality based on fundamental questions of right and wrong?"

1.7 Survey Supplementary Study

For the Supplementary Study, the following questions were asked with both the block order randomized and the questions within each block were also randomized. The three blocks of survey questions (demographics, MFQ-2, Wealth and Inequality) from the main study (listed above) were also you used in the supplementary study. In addition to these, the following three blocks of questions were also asked.

1.7.1 Morality of Excess Beyond Wealth

Below you are going to be asked to rate the morality of having excess in different domains. On a scale from 1 (*Not wrong at all*) to 5 (*Extremely wrong*), Is it morally wrong to have too much.

1. Knowledge
2. Health

3. Compassion
4. Loyalty
5. Authority
6. Equality
7. Wisdom
8. Friends
9. Hair
10. Sexual Activity
11. Happiness
12. Work
13. Eat
14. Environmental Impact
15. Prayer
16. Love for your mother
17. Power
18. Patriotism
19. Arrogance
20. Fun
21. Competitiveness
22. Ambition
23. Anger
24. Water Bottles

1.7.2 Morality of Spending Excessive Wealth

Below you will see a number of different explanations about how extremely rich people gained an excessive amount of wealth. Please rate how moral you find these different paths to wealth acquisition on a scale of (1) Not wrong at all to (5) Extremely wrong.

1. Philanthropy: Some wealthy individuals engage in philanthropic activities, donating significant sums of money to charitable causes, funding research, supporting education initiatives, or establishing foundations to address societal issues. This philanthropy goes above and beyond the tax benefits they could potentially gain.
2. Innovative Impact: Some wealthy individuals choose to invest their money in ventures that aim to create a positive social or environmental impact alongside financial returns. They support businesses focused on sustainability, renewable energy, or social enterprises.
3. Entrepreneurship: Some wealthy individuals can invest their money in starting or supporting new businesses, thereby creating employment opportunities and contributing to economic growth.
4. Arts and Culture: Some wealthy individuals support the arts by investing in art collections, sponsoring exhibitions, or funding cultural institutions to preserve and promote artistic expression.
5. Healthcare and Medical Research: Some wealthy individuals contribute to healthcare advancements by funding medical research, supporting hospitals or clinics, or providing resources for underprivileged communities to access quality healthcare.

6. Lavish Lifestyle: Some wealthy individuals may indulge in lavish lifestyles, spending exorbitant amounts on luxury goods, properties, and services.
7. Lobbying: Some wealthy individuals can use their financial resources to exert influence over political processes, lobbying for policies that align with their personal or business interests, which may not always be in the broader public's best interest.
8. Tax Evasion: Some wealthy individuals may engage in practices to minimize their tax obligations, utilizing offshore accounts or complex financial structures to shield their wealth from taxation.
9. Corruption: Some wealthy individuals use their financial resources to engage in corrupt practices, bribing officials, manipulating regulations, or securing unfair advantages in business dealings.
10. Wealth Management: Some wealthy individuals often engage in extensive estate planning to protect and pass on their assets to future generations.

1.7.3 Morality of Acquiring Excessive Wealth

Below you will see a number of different explanations about how extremely rich people gained an excessive amount of wealth. Please rate how moral you find these different paths to wealth acquisition on a scale of 1) *Not wrong at all* to (5) *Extremely wrong.*

1. Entertainment: Some people gain an excessive amount of wealth through being extremely competent entertainers. For example, extremely successful musicians, actors, comedians, painters, movie directors, or writers.
2. Inheritance: Some people gain an excessive amount of wealth through inheritance, where family members are bestowed their wealth.
3. Genius: Some people gain an excessive amount of wealth through being productive genius revolutionaries. For example, scientists, inventors, or researchers that discover or create a groundbreaking technology that revolutionizes society (e.g. the automobile, internet search engines, the home computer).
4. Luck: Some people gain an excessive amount of wealth through being extremely lucky. For example, they win the lottery or stumble upon valuable assets (e.g. rare paintings at a yard sale).
5. Athletics: Some people gain an excessive amount of wealth through being high-skilled hard-working athletes. For example, extremely successful Olympians, basketball stars, or international soccer players.
6. Fair Production: Some people gain an excessive amount of wealth through exceptional production with fair treatment for the workers. For example, extremely productive companies that have comprehensive benefits packages for their employees, healthy employee relationships, and more equal CEO to average work pay ratio.
7. Government corruption: Some people gain an excessive amount of wealth through government corruption. For example, through embezzlement of public funds, influence peddling and lobbying, or bribery and kickbacks with government officials.
8. Corporate corruption : Some people gain an excessive amount of wealth through corporate corruption. For example, through accounting fraud, embezzlement of company funds, insider trading, bribery and kickbacks, or price fixing and collusion.

9. Nepotism: Some people gain an excessive amount of wealth through nepotism, where family members or friends favor and give preferential treatment over more qualified candidates.
10. Worker Exploitation: Some people gain an excessive amount of wealth through exceptional production with worker exploitation. For example, extremely productive factories where the workers have limited bathroom breaks and the CEO to average worker pay is heavily skewed to the CEO.

References

[1] Arel-Bundock V (2022) modelsummary: Data and model summaries in R. Journal of Statistical Software 103(1):1–23. https://doi.org/10.18637/jss.v103.i01

[2] Atari M, Haidt J, Graham J, et al (2023) Morality beyond the weird: How the nomological network of morality varies across cultures. Journal of Personality and Social Psychology 125(5):1157–1188

[3] Bates D, Mächler M, Bolker B, et al (2015) Fitting linear mixed-effects models using lme4. Journal of Statistical Software 67(1):1–48. https://doi.org/10.18637/jss.v067.i01

[4] Bürkner PC (2017) Brms: An R package for bayesian multilevel models using stan. J Stat Softw 80(1). https://doi.org/10.18637/jss.v080.i01, URL http://dx.doi.org/10.18637/jss.v080.i01

[5] Champely S, Ekstrom C, Dalgaard P, et al (2017) pwr: Basic functions for power analysis

[6] Claessens S, Kyritsis T, Atkinson QD (2023) Cross-national analyses require additional controls to account for the non-independence of nations. Nature Communications 14(1):5776

[7] Global Risk Profile (2022) Data: Global corruption index. GCI Online

[8] J L (2006) Plotrix: a package in the red light district of r. R-News 6(4):8–12

[9] Jr FEH (2023) Harrell Miscellaneous. Https://hbiostat.org/R/Hmisc/

[10] Kuznetsova A, Brockhoff PB, Christensen RHB (2017) lmerTest package: Tests in linear mixed effects models. Journal of Statistical Software 82(13):1–26. https://doi.org/10.18637/jss.v082.i13

[11] Landis J (2022) ggside: Side Grammar Graphics. Https://cran.r-project.org/web/packages/ggside/index.html

[12] Long JA (2019) jtools: Analysis and Presentation of Social Scientific Data. URL https://cran.r-project.org/package=jtools, r package version 2.0.0

[13] Long JA (2019) xtable: Export Tables to LaTeX or HTML. URL https://cran.r-project.org/web/packages/xtable/xtable.pdf, r package version 1.8-4

[14] Lüdecke D (2022) sjPlot: Data Visualization for Statistics in Social Science. URL https://CRAN.R-project.org/package=sjPlot, r package version 2.8.12

[15] Magnusson A, Skaug HJ, Nielsen A, et al (2017) glmmTMB: Generalized Linear Mixed Models using Template Model Builder

[16] McElreath R (2018) Statistical rethinking: A Bayesian course with examples in R and Stan. Chapman and Hall/CRC

[17] Moeinian B (2005) An Easy to Understand Translation of Qur'an Paperback. Lulu

[18] Revelle W (2022) psych: Procedures for Psychological, Psychometric, and Personality Research. Northwestern University, Evanston, Illinois, URL https://CRAN.R-project.org/package=psych, r package version 2.2.9

[19] Rosseel Y (2012) lavaan: An R package for structural equation modeling. Journal of Statistical Software 48(2):1–36. https://doi.org/10.18637/jss.v048.i02

[20] Stanley D, Stanley MD (2018) Package 'apatables'. R Online

[21] Tawney RH (1926) Religion and the Rise of Capitalism. Harcourt Brace, New York

[22] Wei T, Simko V, Levy M, et al (2017) Package 'corrplot'. Statistician 56(316):e24

[23] Wickham H (2016) ggplot2: Elegant Graphics for Data Analysis. Springer-Verlag New York, URL https://ggplot2.tidyverse.org

[24] Wickham H, Bryan J (2023) readxl: Read Excel Files. Https://readxl.tidyverse.org, https://github.com/tidyverse/readxl

[25] Wickham H, Averick M, Bryan J, et al (2019) Welcome to the tidyverse. Journal of Open Source Software 4(43):1686. https://doi.org/10.21105/joss.01686

[26] Wickham H, François R, Henry L, et al (2023) dplyr: A Grammar of Data Manipulation. Https://dplyr.tidyverse.org, https://github.com/tidyverse/dplyr

[27] Wickham H, Miller E, Smith D (2023) haven: Import and Export 'SPSS', 'Stata' and 'SAS' Files. Https://haven.tidyverse.org, https://github.com/tidyverse/haven, https://github.com/WizardMac/ReadStat

[28] Wilke CO (2021) ggridges: Ridgeline Plots in'ggplot2'. URL https://cran.r-project.org/web/packages/xtable/xtable.pdf, r package version R package version 0.5. 2
